# Stage-Specific Transcription Factors Drive Astrogliogenesis by Remodeling Gene Regulatory Landscapes

**DOI:** 10.1016/j.stem.2018.09.008

**Published:** 2018-10-04

**Authors:** Neha Tiwari, Abhijeet Pataskar, Sophie Péron, Sudhir Thakurela, Sanjeeb Kumar Sahu, María Figueres-Oñate, Nicolás Marichal, Laura López-Mascaraque, Vijay K. Tiwari, Benedikt Berninger

**Affiliations:** 1 Institute of Physiological Chemistry, University Medical Center Johannes Gutenberg University Mainz, 55128 Mainz, Germany; 2 Institute of Molecular Biology (IMB), 55128 Mainz, Germany; 3 Broad Institute of MIT and Harvard, Cambridge, MA, USA; 4 Department of Stem Cell and Regenerative Biology, Harvard University, Cambridge, MA, USA; 5 Instituto Cajal, CSIC, Madrid, Spain; 6 Focus Program Translational Neuroscience, Johannes Gutenberg University Mainz, 55131 Mainz, Germany; 7 Centre for Developmental Neurobiology, Institute of Psychiatry, Psychology & Neuroscience, King’s College London, London SE1 1UL, UK; 8 MRC Centre for Neurodevelopmental Disorders, Institute of Psychiatry, Psychology & Neuroscience, King’s College London, London SE1 1UL, UK

**Keywords:** astrocyte, cell fate, epigenetic mechanisms, gene regulation, transcription factors, neural stem cells, astrogliogenesis, neurogenesis

## Abstract

A broad molecular framework of how neural stem cells are specified toward astrocyte fate during brain development has proven elusive. Here we perform comprehensive and integrated transcriptomic and epigenomic analyses to delineate gene regulatory programs that drive the developmental trajectory from mouse embryonic stem cells to astrocytes. We report molecularly distinct phases of astrogliogenesis that exhibit stage- and lineage-specific transcriptomic and epigenetic signatures with unique primed and active chromatin regions, thereby revealing regulatory elements and transcriptional programs underlying astrocyte generation and maturation. By searching for transcription factors that function at these elements, we identified NFIA and ATF3 as drivers of astrocyte differentiation from neural precursor cells while RUNX2 promotes astrocyte maturation. These transcription factors facilitate stage-specific gene expression programs by switching the chromatin state of their target regulatory elements from primed to active. Altogether, these findings provide integrated insights into the genetic and epigenetic mechanisms steering the trajectory of astrogliogenesis.

## Highlights

• Distinct transcriptional programs define consecutive stages of astrogliogenesis
• Stage- and lineage-specific enhancers promote the distinct gene expression programs
• ATF3 and NFIA drive astrocyte differentiation, while RUNX2 promotes astrocyte maturation
• These TFs switch the enhancers from a primed to an active chromatin state

## Graphical Abstract

**Figure:**
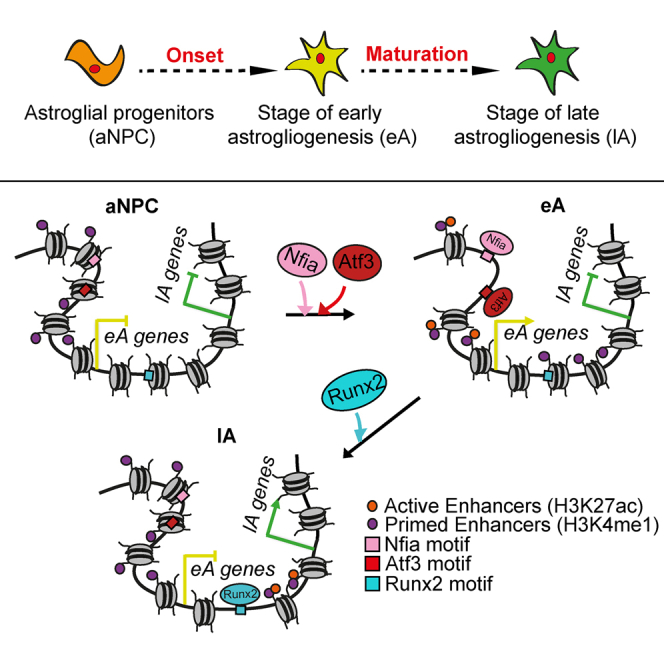

## Teaser

Neural stem cells give rise to both neurons and glia. Modeling gliogenesis *in vitro*, Tiwari et al. show that the generation of astrocytes involves several transcriptionally and epigenetically distinct stages. The authors found that the transcription factors NFIA, ATF3, and RUNX2 play pivotal roles in establishing these stages.

## Introduction

A comprehensive understanding of the molecular mechanisms underlying cellular diversity and cell-fate specification during CNS development remains elusive. In the developing CNS, neural precursor cells (NPCs) are known to give rise first to neurons and then to glia such as astrocytes and oligodendrocytes (Kriegstein and Alvarez-Buylla, 2009). The molecular circuitry underlying the differentiation of NPCs into astrocytes is only beginning to be untangled. Astrocytes are involved in various functions that are important for the establishment, maintenance, and plasticity of the brain (Tiwari and Berninger, 2017). Because of these important roles, malfunctions in astrocytes have been implicated in many neurological diseases (Molofsky et al., 2012).

Despite these advances, the gene regulatory mechanisms that control the differentiation and maturation of astrocytes from NPCs, hereafter referred to as astrogliogenesis, remain to be uncovered. A plethora of signaling pathways are known to play a critical role in the differentiation of NPCs into astrocytes (Kanski et al., 2014). For instance, BMP2 promotes STAT3-mediated astrogliogenesis by forming a STAT3-SMAD1-p300 co-activating complex that initiates expression of astrocyte-specific genes by binding to their promoters (Fukuda et al., 2007, Nakashima et al., 1999). In addition to STAT3 (Bonni et al., 1997, Moon et al., 2002), several other transcription factors (TFs), including SOX9, NFIA (Kang et al., 2012), ETV5/ERM (Li et al., 2012), and ZBTB20 (Nagao et al., 2016), have been implicated as important in astrogliogenesis. Emerging evidence implies that these TFs interplay with epigenetic mechanisms to regulate astrocyte-specific genes. At the onset of astrogliogenesis, STAT3 binds to the Gfap promoter and promotes DNA demethylation, resulting in *gfap* expression (Fan et al., 2005). Similarly, NOTCH signaling induces the expression of Nfia in neural progenitor cells, which then targets the promoters of astrocyte-specific genes and causes DNA demethylation at these promoters by displacing DNMT1 (Namihira et al., 2009). Furthermore, at the onset of gliogenesis, the Polycomb group (PcG) proteins repress pro-neuronal genes, such as *Neurog1*, thereby restricting the neurogenic competence of NPCs (Hirabayashi et al., 2009). Finally, proteins of the high-mobility group nucleosome-binding family, HMGN1, HMGN2, and HMGN3, also play a critical role in promoting differentiation of astrocytes (Nagao et al., 2014). Despite these exciting advances, a comprehensive understanding of the transcriptional and epigenetic mechanisms acting along the differentiation trajectory from NPCs to astrocytes remains elusive. In addition, although the enhancers have a known role in cell-fate specification (Buecker et al., 2014), no studies have performed an in-depth investigation to identify the regulatory elements that are involved in defining the distinct phases of astrogliogenesis and the epigenetic mechanisms and TFs that operate at these sites during this process.

Here we used a model of astrogliogenesis from mouse embryonic stem cells in combination with next-generation sequencing and computational approaches to pinpoint distinct stages along the differentiation trajectory by identifying stage-specific transcriptional programs and epigenetic states. This quest allowed us to discern stage- and lineage-specific regulatory elements that represent putative targets of the TFs Nfia, Atf3, and Runx2.

## Results

### Distinct Gene Expression and Epigenetic Landscape Define Stages of Astrogliogenesis

**Figure 1:**
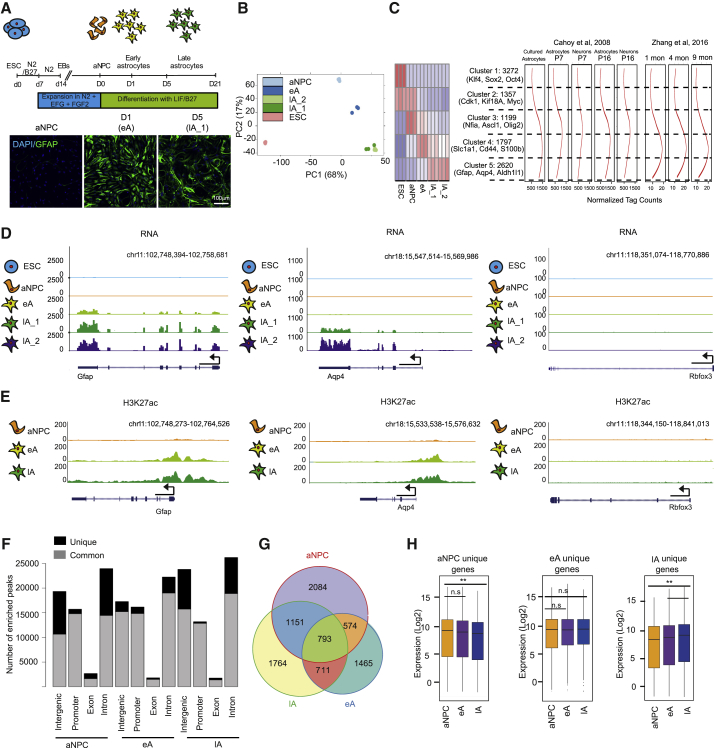
Distinct Gene Expression Programs and Distal Epigenetic Footprints Define Distinct Stages of Astrogliogenesis (A) Schematic representation of astroglial differentiation from ESCs. Immunofluorescence staining of DAPI and GFAP in the ESC-derived astrocytes during phases of astrogliogenesis, including aNPC, eA, lA_1, and lA_2. Scale bar is 100 μm. (B) Principal-component analysis plot depicting the distribution of the transcriptomes of ESC (pink), aNPC (light blue), eA (dark blue), and two stages of lA (shades of green) within the scope of the first two components after RNA-seq in these stages. The x and y axes show the percentage of variance explained by the first 2 principal components. (C) Left side: clustered heatmap depicting the expression of differentially expressed genes during any stage of the *in vitro* astroglial differentiation, along with the names of known key genes in each of these clusters. Expression in the heatmap is scaled from blue (lowest) to red (highest). Right side: locally weighted scatterplot smoothing (lowess) regression line for these clusters in the same order as the published astrocyte transcriptomes by Cahoy et al. (2008) and Zhang et al. (2016). (D and E) University of California Santa Cruz (UCSC) browser track example of expression (D) and H3K27ac enrichment (E) at the astroglial genes, i.e., Gfap and Aqp4, and the neuronal gene Rbfox3 during the stages of the *in vitro* astroglial differentiation (ESC, aNPC, eA, and lA). (F) Stacked-bar plot depicting the number of common and unique (across astroglial differentiation) H3K27ac peaks under each condition of astroglial differentiation sorted by their genomic location, i.e., intergenic, promoter, exon, and intron. (G) Venn diagram representing the overlap of the H3K27ac peaks within a distance of 50 kb from the nearest genes called in aNPC, eA, and lA. (H) Boxplot representing the expression of the genes, as determined as log2-normalized read counts, that are associated with the aNPC, eA, and lA unique H3K27ac peaks depicted in (G).

To investigate the gene regulatory mechanism underlying astrogliogenesis, we first adapted an experimental model that uses mouse embryonic stem cells (ESCs) to generate highly enriched astrogliogenic neural precursor cells (aNPCs) that subsequently differentiate into astrocytes (early astrocyte [eA] and mature or late astrocyte [lA]) (Figure 1A; Figure S1A) (Conti et al., 2005, Pollard et al., 2006). qRT-PCR and immunoblot analyses showed that these astrocytes express established markers (e.g., Gfap, S100b, Nfia, Glast, Aqp4, and Cx43) (Figures S1B and S1C; Figure 1A). Using markers for neuronal cells (TUBB3 and DCX) and progenitor cells (NES, OLIG2, and ASCL1), we found that these cultures exhibit a minimal presence of these cells (Figure S1D). However, we observed differences in the expression levels of AQP4, GLAST, and CX43, indicative of astroglial heterogeneity (Figure S1D). The differentiation of aNPCs into astrocytes was accompanied by a cessation of cell proliferation (Figure S1E). Furthermore, we found that similar to astrocytes *in vivo*, mature astrocytes generated calcium signals upon mechanical stimulation that propagated to neighboring astrocytes, indicative of their functional maturation (Figures S1F and S1G). In line with previous studies, aNPCs were able to generate neurons when leukemia inhibitory factor (LIF) and fetal bovine serum (FBS) were withdrawn from the differentiation medium (Figure S1H) (Conti et al., 2005, Pollard et al., 2006).

To measure the global gene expression changes that accompany astrogliogenesis, we performed high-coverage transcriptome profiling (RNA sequencing [RNA-seq]) of ESCs, aNPCs, and astrocytes at various stages of differentiation, spanning the early and later phases (1, 5, and 21 days, hereafter referred as eA, lA_1, and lA_2, respectively). A principal-component analysis (PCA) of the transcriptome datasets uncovered a progressive separation of the transcription profiles during astrogliogenesis, suggesting that the acquisition of distinct gene expression programs gave rise to stage-specific cellular identity (Figure 1B). The differential expression analysis of the contiguous stages of astrogliogenesis revealed major differences among the early stages, while the stages lA_1 and lA_2 were transcriptionally similar, indicating that terminal astroglial gene expression was largely established at lA_1 (Figure 1B). Thus, in the subsequent analysis, we focused on the mature or late astrocytes, hereafter referred to as lA. We then identified genes that were differentially expressed using pairwise comparisons of subsequent stages during astrogliogenesis (Figure S1I; Table S1) and grouped them into five clusters (Figure 1C; Table S2), in temporal order, according to their expression kinetics during astroglial differentiation. Moreover, genes that were highly expressed during astrogliogenesis from mouse ESCs were highly expressed in astrocytes *in vivo* (Cahoy et al., 2008, Zhang et al., 2016) and at higher levels than those found in neurons (Figure 1C). This analysis showed progressive changes in the expression profiles and reflected the developmental trajectory as supported by gene ontology (GO) term analysis of these gene clusters, showing enrichment of stage-relevant biological pathways (Table S2). Cluster 1 (ESC genes) and cluster 2 (ESC/aNPC genes) were enriched with genes involved in cell-cycle and metabolic pathways, reflecting the proliferative activity of these cell types and the associated high metabolic rate (Table S2; Figure 1C; Figures S1J and S1K). Cluster 3 (aNPC/eA) and cluster 4 (eA) showed enrichment with genes involved in nervous system development, reflecting the ongoing process of astrogliogenesis (Table S2; Figure 1C; Figures S1L and S1M). Cluster 5 (lA_1 and lA_2) was enriched with GO terms such as signaling and cytokine response, which are features that have been linked to mature astrocytes (Michelucci et al., 2016) (Table S2; Figure 1C; Figure S1N). Consistent with the progressive differentiation and maturation of astrocytes, we found that Gfap was induced at the onset of astroglial differentiation (eA), while the maturation marker Aqp4 only appeared during later stages (Figure 1D). In contrast, the mature neuronal marker Rbfox3 was never expressed during astrogliogenesis (Figure 1D). In addition, a comparison of our datasets with the astrocyte gene expression data retrieved from Network Glia (http://www.networkglia.eu), which includes published datasets (Cahoy et al., 2008), revealed similar expression kinetics in signature genes of *in vivo*- and *in vitro*-differentiated astrocytes (Figure S1O). Altogether, these features confirm that our *in vitro* astrogliogenesis model is highly suitable for studying gene regulatory mechanisms underlying astrocyte differentiation. Furthermore, our observations clearly highlight that the consecutive stages of astrogliogenesis can be defined by distinct gene expression profiles.

Acetylation of lysine 27 at histone H3 (H3K27ac) marks active proximal and distal regulatory elements (Creyghton et al., 2010, Shlyueva et al., 2014). To identify these regulatory regions, we generated H3K27ac chromatin immunoprecipitation sequencing (ChIP-seq) profiles during the stages of astrogliogenesis (aNPC, eA, and lA) (Figure S1P). Single-gene visualizations showed an expected enrichment in H3K27ac levels at astroglial genes (e.g., Gfap and Aqp4), while neuronal genes (e.g., Rbfox3) failed to show enrichment of this mark during astrogliogenesis (Figure 1E). Although the total number of H3K27ac-enriched sites remained in the same range for all stages, they exhibited a substantial number of uniquely acquired H3K27ac-enriched regions. These uniquely enriched sites were particularly conspicuous at the aNPC and lA stages, whereas astrocytes at the eA stage showed far fewer unique H3K27ac-enriched sites, possibly reflecting an intermediate epigenetic profile at this transitory stage (Figures S1Q and S1R).

The genomic distribution of H3K27ac-enriched sites revealed its very similar occurrence at promoters and non-promoter (or ‘distal’, including intergenic, exons, and introns) regions in aNPC, eA, and lA stages (Figure 1F). However, the stage-specific H3K27ac sites were predominantly located in non-promoter regions, except for eA (Figure 1F). We also validated that the H3K27ac enrichment at promoter and distal regions of several known astrocyte genes increased during astroglial differentiation using ChIP-qPCR experiments (Figures S1S and S1T). To identify genes putatively regulated by the distal H3K27ac sites, we assigned genes according to proximity (−50 kb) to the stage-specific distal H3K27ac sites. Although many of these sites shared the same nearest gene, many genes were uniquely associated with stage-specific H3K27ac-enriched sites (Figure 1G). These non-overlapping genes were expressed at significantly higher levels at their corresponding stages, except for the eA stage (Figure 1H).

### Epigenetic Priming Precedes Stage-Specific Acquisition of Active Chromatin at Regulatory Regions during Astrogliogenesis

**Figure 2:**
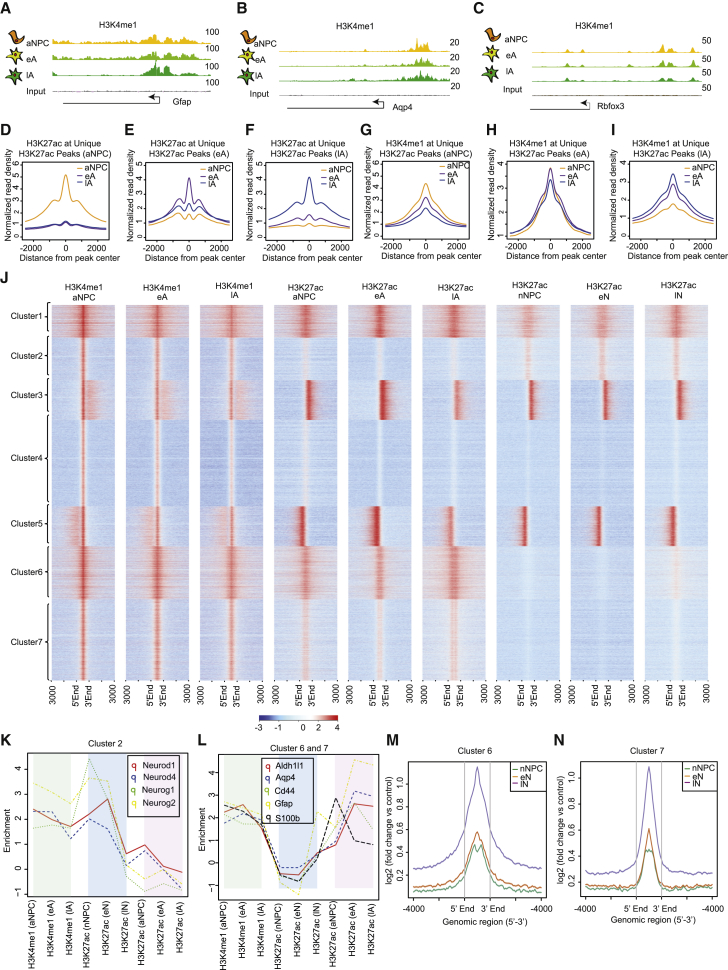
Epigenetic Priming of Lineage- and Stage-Specific Regulatory Elements before Their Activation during Astrogliogenesis (A–C) UCSC browser track showing H3K4me1 enrichment at the regulatory regions of astroglial genes (A: Gfap, B: Aqp4) and neuronal gene (C: Rbfox3). (D–I) Profile plots depicting the enrichment of H3K27ac (D–F) and H3K4me1 (G–I) at the aNPC (D and G), eA (E and H), and lA (F and I) unique H3K27ac peaks. (J) Unsupervised clustering-based heatmap depicting the ChIP-seq enrichment of H3K4me1 during all stages of astrogliogenesis and H3K27ac during all stages of astrogliogenesis and neurogenesis at the H3K4me1 peaks in aNPC. (K and L) Line plot depicting the enrichment of H3K4me1 during all stages of astrogliogenesis and H3K27ac during all stages of astrogliogenesis and neurogenesis in the regulatory regions of neuronal marker genes (K) and astroglial marker genes (L) that belong to clusters 2 and 6 or 7 from (J), respectively. (M and N) H3K4me1 enrichment during neuronal differentiation at cluster 6 (M) and cluster 7 (N) sites.

Mono-methylation of lysine 4 at histone H3 (H3K4me1) marks primed or active enhancers in the absence or presence of H3K27ac, respectively (Creyghton et al., 2010). Given the critical role of the crosstalk among various chromatin marks at regulatory elements in defining cell-type-specific gene expression programs, we investigated whether H3K27ac enrichment is related to the occurrence of H3K4me1 at these sites during astrogliogenesis. Toward this end, we generated H3K4me1 ChIP-seq profiles during the stages of astrogliogenesis (aNPC, eA, and lA) (Figure S2A). Further analysis revealed a substantial number of stage-specific H3K4me1-enriched regions, which were nearly three-fold higher in lA (Figure S2B). The genes with H3K4me1-enriched promoters included Gfap and Aqp4, which already exhibited high levels of H3K4me1 in aNPC before acquisition of H3K27ac (Figure 1E) and transcription (Figures 2A and 2B) at the eA and lA stages, respectively, indicative of priming. High levels of H3K4me1 were also detected at the promoters of neurogenic genes, such as Rbfox3 in aNPC (Figure 2C). These H3K4me1-enriched regions were not only at the promoters of astrocyte-specific genes but also at the distal regions, as validated by ChIP-qPCR assays (Figures S2C and S2D). Astrocyte-specific genes were either not primed or minimally primed in ESCs, and they acquired priming only upon commitment to the astrocyte lineage, i.e., in astrocyte progenitor cells (aNPCs) (Figures S2C and S2D).

We next investigated the H3K4me1 enrichment patterns at the stage-specific H3K27ac sites during astrogliogenesis. A profile plot depiction of these H3K27ac sites revealed their stage-specific high enrichment during astrogliogenesis (Figures 2D–2F). The aNPC-specific H3K27ac sites showed the highest enrichment of H3K4me1 at the aNPC state, and upon astrocyte differentiation, H3K27ac enrichment was lost in these regions and H3K4me1 levels were reduced (Figures 2D and 2G). eA-specific H3K27ac sites were already marked by H3K4me1 in aNPC, and H3K4me1 enrichment patterns were maintained through the eA stage while gaining H3K27ac (Figures 2E and 2H). Furthermore, these regions lost H3K27ac at the lA stage, and the levels of H3K4me1 were reduced. Similarly, lA-specific H3K27ac sites gained H3K4me1 at the eA stage and increased this mark at the lA stage (Figures 2F and 2I). These data suggest that epigenetic priming of astroglial genes occurs before their acquisition of an active chromatin state and transcriptional activation.

We then attempted to unravel the distinct set of genes that show different dynamics of H3K27ac at the H3K4me1 primed sites during astrogliogenesis versus neurogenesis and extended these analyses to reveal the gene regulatory landscapes that contribute to the divergence of these two lineages. Therefore, we adapted an established system in which ESCs differentiate into neurogenic neural precursor cells (nNPCs) and subsequently into terminally differentiated neurons (TNs) via different stages (early neuron [eN] and late neuron [lN]) (Bibel et al., 2004) (Figure S2E). Previous studies have shown distinct epigenomic and transcriptomic changes in this model of neurogenesis that closely mimicked *in vivo* (Mohn et al., 2008, Stadler et al., 2011, Thakurela et al., 2015). A clustering analysis of the regions primed in aNPC produced seven sets of patterns (Figure 2J; Figure S2F; Table S3). Clusters 1, 3, and 5 showed minimal dynamics of H3K4me1 and H3K27ac during both astrogliogenesis and neurogenesis. Cluster 2 represented regions primed in aNPC that gain H3K27ac only during neurogenesis. Further analysis of cluster 2 showed that this cluster contains genes that are specific to neuronal differentiation, such as NeuroD1, Neurog1, and Neurog2 (Figure 2K), supporting multipotency at the aNPC stage. Cluster 4 contained genes that are marked by H3K4me1 without subsequent enrichment of H3K27ac in either lineage. Finally, clusters 6 and 7 contained sites that are primed in aNPC and gain H3K27ac specifically during astrogliogenesis. These clusters comprise astrocyte-specific genes, such as Gfap, Aqp4, Aldh1l1, Aldoc, Cd44, and S100b (Figure 2L). The switch from a primed to an active state is accompanied by a transcriptional induction of genes in the nearest to these sites (Figure S2G). GO analysis of cluster 2 showed enrichment of genes related to neuronal differentiation (Figure S2H), whereas cluster 6- and cluster 7-specific genes were related to cell signaling and cell morphogenesis (Figure S2I). Furthermore, cluster 6- and cluster 7-contained sites were primed in nNPC, eN, and lN, whereas they never gained H3K27ac during neuronal differentiation (Figures 2M and 2N). Altogether, these data reveal epigenetic priming in regulatory elements, which precedes the stage-specific acquisition of active chromatin and transcriptional activation during astrogliogenesis.

### Distinct TFs Shape the Epigenetic Landscape to Demarcate Astrocyte versus Neuronal Fate

Prompted by our finding that these regulatory elements are dynamically used during astrogliogenesis, we were interested in identifying TFs that function at these elements and how they differ from the factors that orchestrate neurogenesis. Using datasets from the neuronal differentiation model described earlier (Bibel et al., 2004, Thakurela et al., 2015), our analysis revealed strikingly different gene expression programs during astrocytic differentiation compared to neuronal differentiation (Figure S3A) and a large number of genes differentially expressed for aNPC versus nNPC, eA versus eN, and lA versus lN (Figures S3B–S3D). GO analysis revealed that highly induced genes in aNPC were enriched for nervous system development, including gliogenesis (aNPC versus nNPC) (Figure S3E). Furthermore, the genes highly upregulated in eAs compared to eNs were enriched for proliferation-, migration-, and cell adhesion-related genes (Figure S3F). Finally, the genes that were more higher expressed in lAs versus lNs were enriched with functions such as signaling and cytokines (Figure S3G). In addition to the TFs and epigenetic regulators (ERs) known to be relevant for astrogliogenesis, such as Nfia (Deneen et al., 2006, Piper et al., 2010), this list contained several TFs and ERs whose function in astrogliogenesis is unknown (Figures S3H–S3M; Table S1).

**Figure 3:**
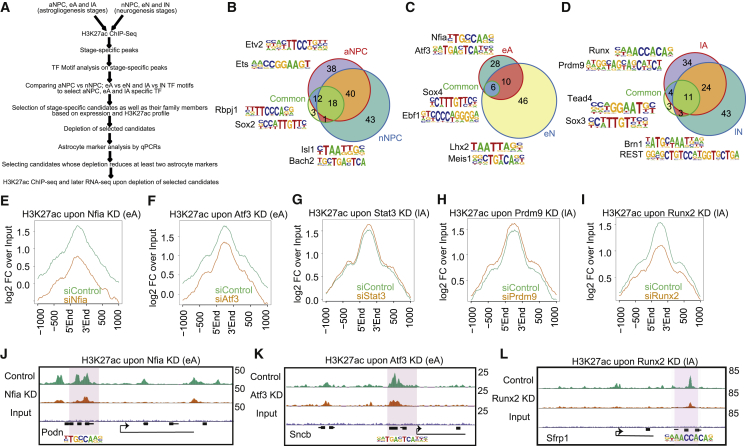
Distinct TFs Set Up the Active Distal Regulatory Landscape Underlying Astroglial Differentiation (A) Workflow that includes the outline of the computational pipeline for how and which TFs were selected, as well as the follow-up experimental part, including knockdown of these TFs and accompanied phenotypic and/or marker analysis. (C–D) Venn diagram depicting the overlap of motifs that are enriched at unique H3K27ac peaks, including promoters, in aNPC and nNPC (A), eA and eN (B), and lA and lN (C). Shown inset are two representative motifs from each set. (E–I) Profile plots representing H3K27ac enrichment at the genomic location of the motifs of the respective TFs within the scope of the unique H3K27ac peaks in eA (Nfia and Atf3 in E and F) and lA (Stat3, Prdm9, and Runx2 in G–I) upon depletion of TFs. The enrichment is depicted as a log two-fold change above the genomic input. (J–L) UCSC browser track example from (E), (F), and (I).

To reveal the differential chromatin landscape during astroglial versus neuronal differentiation, we compared the uniquely enriched H3K27ac sites during various stages of astrogliogenesis to those of the corresponding stages of neurogenesis (Figure 3A; Figure S3N). This comparison revealed that stage-specific H3K27ac-enriched regions were largely unique to this lineage and did not occur during neurogenesis (Figure S3N). Similar findings were observed in comparisons with tissues from other lineages (Figures S3O and S3P). We then investigated whether H3K27ac-enriched genomic regions during astrogliogenesis recruit TFs that are similar to or different from those that are recruited during neurogenesis. Thus, we performed a motif enrichment analysis of the unique H3K27ac peaks during each stage of astrogliogenesis, compared these peaks with those that occurred during the corresponding stages of neurogenesis, and extended similar analysis to common peaks (Figure 3A). Although several enriched TF motifs were common between the astrogliogenesis and the neurogenesis stages, a remarkable number of motifs were unique to the astrocyte or the neuronal differentiation stages (Figures 3B–3D; Figure S3Q; Table S4). These motifs included binding sites for many TFs that have previously been shown to be critical for astrogliogenesis (e.g., Nfia and Runx2) and neurogenesis (e.g., Lhx2 and Brn1) (Table S4) (Deneen et al., 2006, Dominguez et al., 2013, Okawa et al., 2016, Subramanian et al., 2011). This analysis revealed several additional TFs that have not been previously implicated in astrogliogenesis and neurogenesis, thus warranting further investigation.

TFs that function at distal regulatory elements often contribute to the activation of these sites (Shlyueva et al., 2014, Thakurela et al., 2015). Therefore, we investigated whether the TFs that were predicted to be enriched at the active regulatory elements during astrogliogenesis contribute to the active chromatin state in these regions (Figure 3A; Figure S3Q). Thus, we performed small interfering RNA (siRNA)-mediated depletion of selected stage-specific TFs during the eA and lA stages and shortlisted the TFs whose knockdown impaired the acquisition of astrocyte markers (Figure 3A; Figures S3R–S3T). This approach led to the identification of Nfia and Atf3 at the eA stage and Stat3, Runx2, and Prdm9 at the lA stage of astrogliogenesis (Figure 3A; Figures S3R–S3U). While Nfia was expressed during each stage of astroglial differentiation, Atf3 and Stat3 are specifically upregulated in eA and lA, respectively (Figures S3V and S3W). In contrast, Runx2 (Figures S3V and S3X) and Prdm9 showed highest expression in lA and aNPC, respectively (Figure S3V). However, while Nfia, Atf3, and Stat3 transcription was low during neurogenesis, Prdm9 and Runx2 showed higher expression (Figure S3V). We then investigated whether knockdown-induced defects in the astrocytic gene expression were due to an aberrant epigenetic state at the target distal regulatory elements. Therefore, we assessed the levels of H3K27ac at putative target sites using ChIP-seq analysis after their depletion during astrogliogenesis. The loss of Nfia and Atf3 in eA and the loss of Runx2 in lA led to a reduction in the H3K27ac levels at putative distal target sites, while the depletion of the other TFs (Stat3 and Prdm9) did not have such an effect (Figures 3E–3I). These global observations were validated by single-locus visualizations (Figures 3J–3L). In summary, these findings reveal lineage-specific TFs that shape the epigenetic landscape of distal regulatory elements during astrocyte differentiation and maturation.

### Stage- and Lineage-Specific Activation of Regulatory Elements during Astrogliogenesis and Neurogenesis

**Figure 4:**
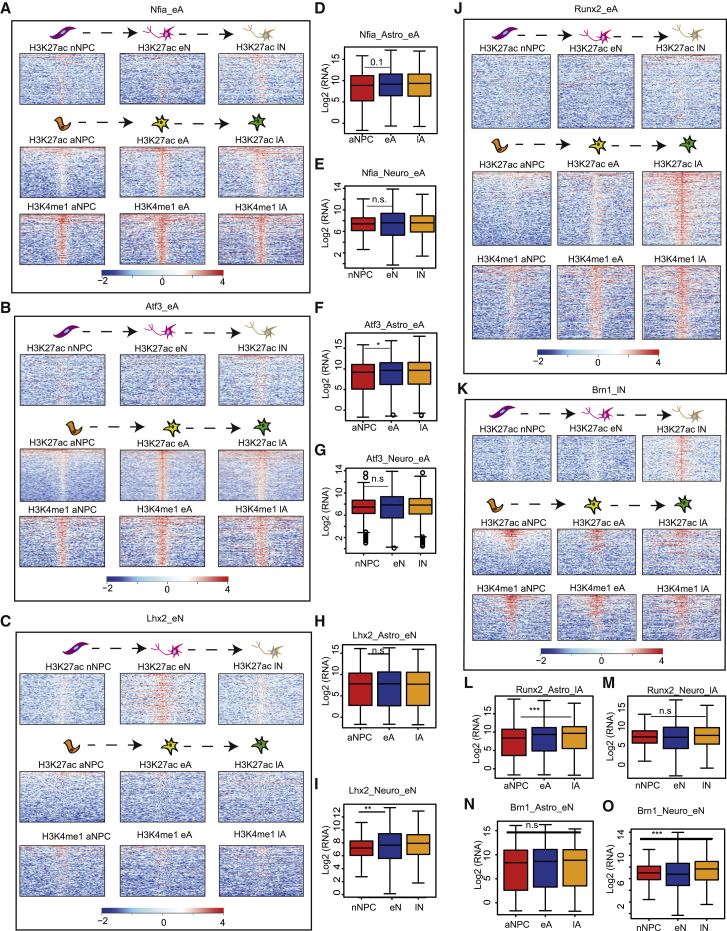
Distinct TF-Target Regulatory Elements Are Stage- and Lineage-Specifically Activated during Astrogliogenesis versus Neurogenesis (A–C) Heatmaps depicting the enrichment of H3K27ac and H3K4me1 during stages of either astroglial or neuronal differentiation at the genomic locations of the Nfia (A), Atf3 (B), and Lhx2 (C) motifs within the scope of unique eA, in the cases of Nfia and Atf3, and eN, in the case of the Lhx2, H3K27ac peaks, including the promoters. (D–I) Expression of the genes nearest to and within 50 kb of the sites depicted in (A)–(C) in astroglial (D, F, and H) and neuronal (E, G, and I) differentiation (log2-normalized read counts). (J and K) Same as (A)–(C) except for the Runx2 and Brn1 motif sites within the scope of the lA and lN H3K27ac unique peaks, respectively, including the promoter. (L–O) Same as (D)–(I) except for the Runx2 and Brn1 motif sites within the scope of the lA and lN unique H3K27ac peaks, respectively, including the promoter.

Prompted by our findings that distinct TFs are required for conferring an active chromatin state to distal regulatory elements, we next investigated the activation dynamics of the TF-target elements during astrogliogenesis and neurogenesis by measuring H3K27ac levels specifically at these sites. Putative Nfia and Atf3 sites were highly enriched with H3K27ac only during the eA stage of astrogliogenesis; these sites remained devoid of H3K27ac during neurogenesis (Figures 4A and 4B; Figures S4A–S4F). Conversely, a similar analysis of the eN-specific TF Lhx2 showed that its putative target sites gain H3K27ac in eN, but not in eA (Figure 4C; Figures S4G–S4I). The expression of the genes nearest to these sites also tended to be higher in eA compared to that in eN for both Nfia and Atf3, while in the case of Lhx2, the nearest genes were induced in eN, but not in eA (Figures 4D–4I). Similarly, putative Runx2 and Brn1 sites showed elevated H3K27ac only in lA and lN, respectively (Figures 4J and 4K; Figures S4J–S4O). In keeping with this, the genes nearest to these Runx2 and Brn1 sites were significantly induced in IA and lN, respectively (Figures 4L–4O). In addition, by comparing the sites putatively occupied by Nfia, Atf3, and Runx2 at all H3K27ac peaks throughout astrogliogenesis, we observed eA stage-specific H3K27ac enrichment at Nfia and Atf3 motifs and lA stage-specific H3K27ac enrichment at the Runx2 motif (Figure S4P). Consistent with our previous observations, Nfia, Atf3, and Runx2 sites were already primed during the preceding stage, before they acquired H3K27ac in eA and lA, respectively (Figures 4A, 4B, and 4J). Altogether, our data suggest that Nfia, Atf3, and Runx2 are crucial for switching the chromatin state of their putative target elements from primed to active, thereby driving the gene expression program underlying astrocyte differentiation and maturation.

### Nfia, Atf3, and Runx2 Mediate Gene Expression Programs Underlying Astrogliogenesis

**Figure 5:**
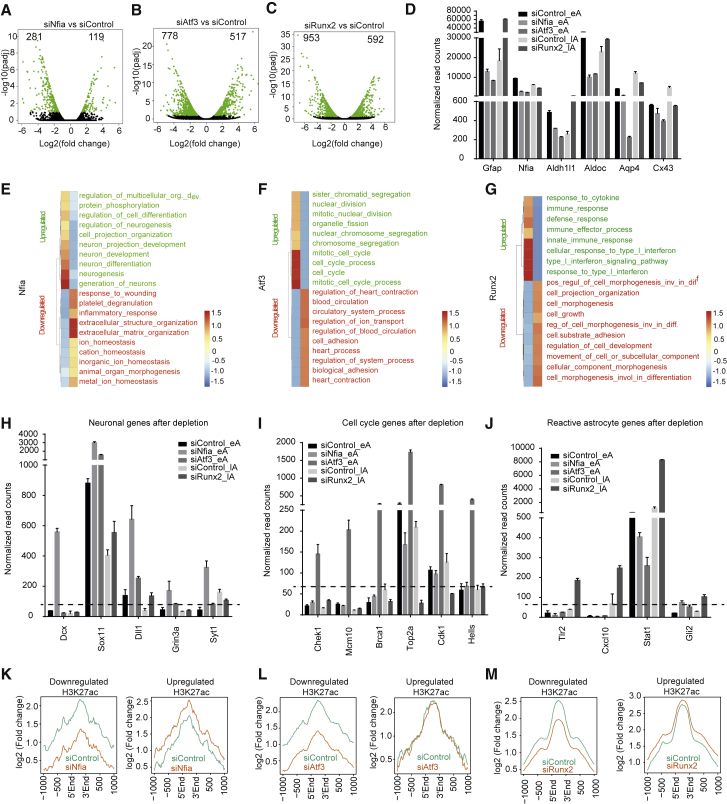
Astroglial TFs Regulate Distinct Gene Expression Programs during Astrogliogenesis (A–C) Volcano plots depicting the differential expression of genes between the control conditions and the conditions with depletion of Nfia in eA (A), Atf3 in eA (B), and Runx2 in lA (C) (green, up- and downregulated; black, no change). (D) Bar plots representing the expression of astroglial markers (Gfap, Nfia, Aldh1l1, Aldoc, Aqp4, and Cx43) under the control and depleted conditions. Values are plotted as normalized read counts. (E–G) Heatmap depicting the top gene ontologies enriched with downregulated (marked in red) and upregulated (marked in green) genes upon Nfia knockdown (E) and Atf3 knockdown (F) in eA and Runx2 knockdown (G) in lA. (H–J) Examples of genes upregulated upon depletion of Nfia (H), Atf3 (I), and Runx2 (J). (K–M) Profile plot of H3K27ac enrichment under the control conditions and the conditions with depleted Nfia (K), Atf3 (L), and Runx2 (M) in the regulatory regions (H3K27ac peaks near deregulated genes) of the upregulated genes and downregulated genes upon Nfia and Atf3 reduction in eA and Runx2 reduction in lA.

To further investigate the target genes that are under the direct transcriptional control of Nfia, Atf3, and Runx2, we performed transcriptome profiling (RNA-seq) after performing an siRNA-mediated depletion of these TFs at the eA stage (Nfia and Atf3) or the lA stage (Runx2). Our analysis revealed a large number of differentially expressed genes after their depletion (Figures 5A–5C; Table S1). Depletion of Nfia and Atf3 led to changes in astrocyte morphology, as well as downregulation in astroglial markers, such as Gfap, Aldh1l1, Aldoc, and Aqp4, while Runx2 knockdown resulted in the downregulation of genes such as Nfia, Cx43, and Aqp4, but not of other markers, such as Gfap, which was even upregulated, suggesting a potential role for Runx2 in preventing a reactive state (Figure 5D; Figures S5A and S5B). While Nfia depletion had no effect on proliferation, apoptosis, or cell cycle (Figures S5C–S5E), Atf3 and Runx2 knockdown reduced apoptosis (Figures S5C–S5E) and Runx2-depleted cells exhibited G0/G1 arrest (Figures S5C–S5E).

The genes that were downregulated or upregulated after the Nfia depletion exhibited enrichment in extracellular matrix organization- and neuronal differentiation-related GO, respectively, suggesting that Nfia may repress the neurogenesis-related program (Figure 5E). However, genes that were downregulated or upregulated after the Atf3 knockdown were enriched with terms related to cell adhesion or mitotic cell-cycle processes, respectively, implying that these genes are relevant for exiting the cell cycle and entering astrocyte differentiation (Figure 5F). In contrast, after Runx2 depletion, the downregulated genes were enriched with GO terms related to cell morphogenesis and differentiation, while the upregulated genes were enriched with genes related to cytokine response, suggesting that Runx2 promotes differentiation and may counteract acquisition of a reactive phenotype (Figure 5G). Single-gene examples substantiated our observations that Nfia and Atf3 promote astrogliogenesis by suppressing neurogenesis and promoting cell-cycle exit of progenitors, respectively, while Runx2 counteracts activation of a reactive phenotype to promote astrocyte maturation (Figures 5H–5J). Genes that were downregulated upon Nfia, Atf3, and Runx2 depletion were upregulated during astrogliogenesis, corroborating the specific role of these TFs in astrogliogenesis (Figures S5F–S5H). In addition, the genes downregulated upon Nfia and Atf3 knockdown are expressed at a higher level in astrocytes compared to neurons, and an opposite pattern was observed for the upregulated genes (Figures S5I and S5J). We did not observe differential expression in astrocytes versus neurons in the case of Runx2-deregulated genes (Figure S5K).

To determine whether the regulatory landscape of the deregulated genes is also altered following TF depletion, we assessed H3K27ac enrichment at stage-specific regulatory sites nearest to these genes (Atf3 and Nfia for eA and Runx2 for lA). In each case, downregulated genes lost H3K27ac enrichment upon knockdown of these factors, indicating that these TFs have an activating function at these sites (Figures 5K–5M). Strikingly, in the case of upregulated genes, H3K27ac enrichment at their regulatory sites did not change for Atf3 and Runx2 and only slightly increased for Nfia, suggesting that the activation of these genes was likely a result of secondary cascades. Overall, our data suggest that Nfia, Atf3, and Runx2 are critical for mediating the gene expression programs underlying astrogliogenesis.

### Nfia, Atf3, and Runx2 Directly Bind Predicted Target Elements and Induce Chromatin Accessibility

**Figure 6:**
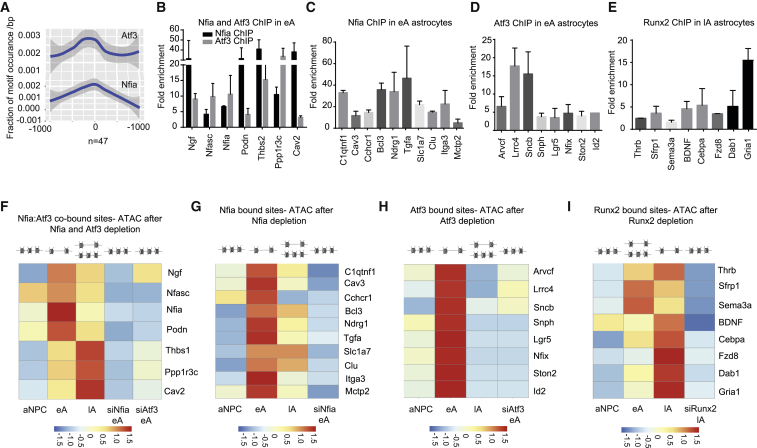
Nfia, Atf3, and Runx2 Directly Bind Predicted Target Elements and Induce Chromatin Accessibility (A) Motif enrichment analysis of the set of commonly deregulated and H3K27ac-enriched genes (47 genes) upon Nfia and Atf3 depletion. (B–E) ChIP-qPCR following Nfia, Atf3, and Runx2 ChIP to detect the Nfia and Atf3 co-occupied (B), Nfia (C), Atf3 (D), and Runx2 (E) binding at predicted Nfia, Atf3, and Runx2 motif sites in the candidate regulatory regions. Enrichments are plotted as a ratio of precipitated DNA (bound) to total input DNA and then further normalized to an intergenic region. (F–I) qPCR following ATAC in aNPC, eA, and lA and after Atf3 and Nfia (F; eA), Nfia (G; eA), Atf3 (H; eA), and Runx2 (I; lA) depletion at regulatory regions listed in (B)–(E), depicted as a heatmap. Enrichments are plotted as in (B)-(E).

Given the known cooperativity among TFs that control cell-fate decisions, we assessed whether Nfia and Atf3 collaborate to regulate early astrogliogenesis. A comparison of the transcriptome data after Nfia and Atf3 knockdown revealed a significant overlap among the genes that were downregulated after their depletion (n = 198 genes), suggesting similar aberrations in the astrogliogenesis program after their knockdown (Figure S6A; Table S5). Based on H3K27ac enrichment, a significant set of genes that were commonly downregulated after Nfia and Atf3 depletion exhibited binding sites for both TFs (n = 47/198), suggesting their potential direct cooperativity during astrocyte differentiation (Figure 6A; Figure S6A).

Given our findings that Nfia, Atf3, and Runx2 are crucial for switching the chromatin state of distal regulatory elements from primed to active, we next investigated whether these factors bind the predicted target elements at the respective stages of astrogliogenesis. Toward this end, we reanalyzed our datasets to validate the reduction in expression and H3K27ac enrichment at selected target genomic loci following depletion of these TFs (Figures S6B–S6I) Next, ChIP assays for Nfia and Atf3 in eA and Runx2 in lA to demonstrated binding at the selected target sites (Figures 6B–6E).

Finally, we investigated whether the target sites of these TFs gain in accessibility while they are bound by these TFs and whether this increase in accessibility depends on the presence of these factors. Assay for transposase-accessible chromatin (ATAC) showed that these sites become more open in the corresponding stages concomitant to TF binding and acquisition of H3K27ac (Figures 6F–6I; Figures S6J–S6M). Conversely, following depletion of these TFs, these target sites showed a significant reduction in chromatin accessibility (Figures 6F–6I; Figures S6N–S6Q). Together with our previous observations, these data support the notion that Nfia, Atf3, and Runx2 directly target distal regulatory elements and induce their active state, as manifested by increased chromatin accessibility, to drive gene expression programs underlying the differentiation and maturation of astrocytes.

### Nfia, Atf3, or Runx2 Overexpression Steers Neurogenic Radial Glia Away from Generating Neurons and Promotes Astroglial Progenitor Generation *In Vivo*

**Figure 7:**
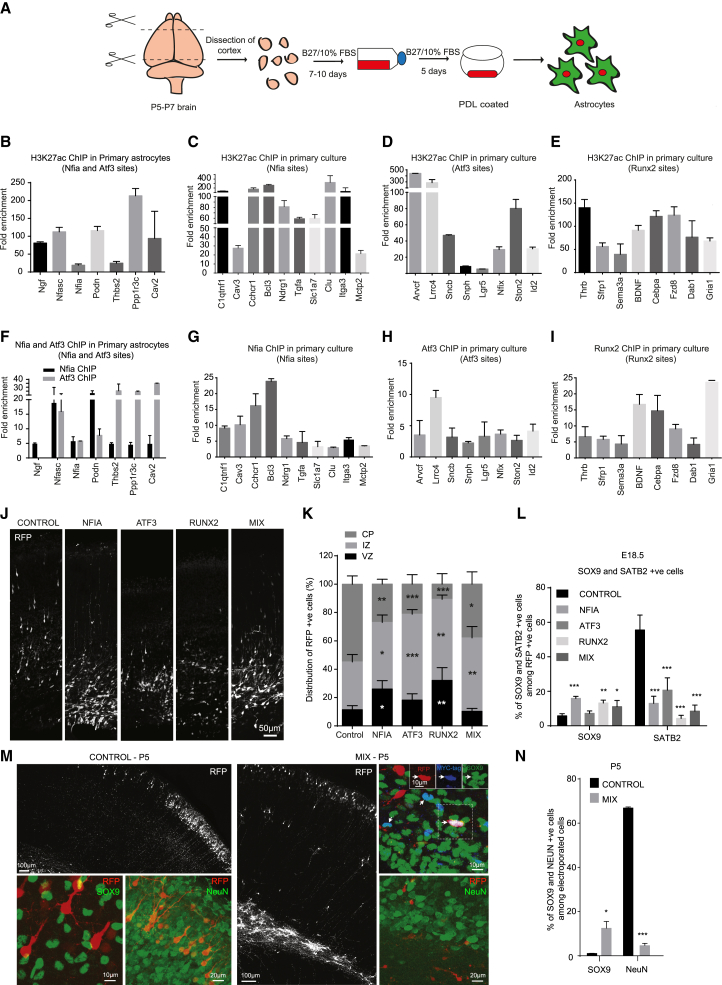
Astroglial TFs Regulate a Similar Set of Sites in Primary Cultures, and Their Overexpression *In Vivo* Alters the Fate of Cortical Progenitors (A) Schematic representation of primary astrocyte culture. (B–E) ChIP-qPCR following H3K27ac ChIP in primary astrocytes derived from P5 mice to detect its enrichment at the Nfia and Atf3 co-occupied (B), Nfia (C), Atf3 (D), and Runx2 (E) motif sites in the candidate regulatory regions. Enrichments are plotted as a ratio of precipitated DNA (bound) to total input DNA and then further normalized to an intergenic region. (F–I) ChIP-qPCR following Nfia (F and G), Atf3 (F and H), and Runx2 (I) ChIP in primary astrocytes derived from P5 mice, showing their occupancy. Enrichments are plotted as in B-E. (J) Distribution of electroporated cells at the E18.5 stage upon *in utero* electroporation of E15.5 ventricular and/or subventricular progenitors using a control pCIDRE plasmid or pCIDRE plasmid encoding NFIA, ATF3, RUNX2, or a combination of all three factors (MIX) (revealed by RFP immunolabeling, shown as white cells). (K) Quantification of (J). (L) Percentage of SOX9- and SATB2-positive cells among RFP-positive cells from cortices electroporated with pCIDRE control or pCIDRE-TF plasmids (single TFs or in combination) and analyzed at the E18.5 stage. (M) Tilescan images showing distribution of electroporated cells at the P5 stage following *in utero* electroporation as above in control or MIX condition (tagged with MYC). Representative images of SOX9, MYC, and NeuN immunofluorescent staining in control and MIX conditions. (N) Quantification of SOX9 and NeuN in electroporated cells in the MIX condition. MYC was used to quantify the expression of SOX9 in electroporated cells in the MIX condition.

To corroborate our key observations on astrogliogenesis from ESCs, we employed primary cortical astrocytes from postnatal day 5 (P5) mice to analyze occupancy by the astrogliogenic TFs identified here and the occurrence of active chromatin landscape at defined target sites (Figure 7A). Primary astrocytes exhibited high levels of H3K27ac at the identified TF-target regulatory elements (Figures 7B–7E). Further analysis using ChIP assays for Nfia, Atf3, and Runx2 confirmed the occupancy by these TFs at their predicted sites (Figures 7F–7I).

Given that these TFs were found to function in activation of astroglial-specific regulatory elements but were not engaged in activation of similar elements in neurogenesis, we hypothesized that the expression of these factors might interfere with the execution of neurogenesis programs during embryonic development. We thus performed *in utero* electroporation (IUE) of mouse cortical progenitors on embryonic day (E) 15.5 (Figueres-Oñate et al., 2016, Pataskar et al., 2016) with plasmids encoding NFIA, ATF3, or RUNX2 or a mixture of these three TFs (MIX) (Figures S7A and S7B). The successful expression of the TFs was confirmed in HEK293T cells by immunoblotting (Figure S7C). At E18.5, cells electroporated with the control plasmid exhibited a typical distribution in the developing cortex that is characterized by migration to the cortical plate (CP) (54.8% ± 5.8% of red fluorescent protein (RFP)-positive cells in the CP, n = 3 animals) (Figures 7J and 7K). In contrast, most cells overexpressing each of the astrogliogenic TFs or their mixture failed to populate the CP (Figures 7J and 7K). Instead, these cells were retained in the GLAST-positive progenitor zone (Figures 7J and 7K; Figure S7D; Table S6). Furthermore, to evaluate whether the cells ectopically expressing these TFs are prevented from acquiring a neurogenic fate, we analyzed the expression of SATB2, a marker of upper-layer neurons generated at this embryonic stage, in electroporated cells (Figure 7L; Figure S7E; Table S6). This analysis demonstrated that cells expressing any of these TFs alone or in combination fail to gain expression of SATB2, in line with their retention below the CP. Further analysis of the expression of the astroglial progenitor marker SOX9 in electroporated cells showed that the expression of these TFs causes neurogenic radial glia to generate a larger proportion of SOX9-positive progenitors (Figure 7L; Table S6).

To characterize the influence of the TFs at a later stage of development, we performed *in utero* electroporation in E15.5 cortical progenitors using MIX and analyzed at the P5 stage. Consistent with our results at E18.5, TF-overexpressing cells were mostly retained in the progenitor zone at P5, although most control-electroporated cells generated neuronal cells that already populated the upper cortical layers (Figure 7M). Moreover, we observed a significant increase in the number of SOX9-positive cells among TF-electroporated cells compared to control (Figures 7M and 7N; Table S6). In contrast, a converse pattern was observed for the mature neuronal marker NeuN (Figures 7M and 7N; Table S6). Altogether, these results suggest an increase in the generation of astroglial progenitors at the expense of neurons.

## Discussion

We aimed to identify key regulators of astrogliogenesis based on lineage- and stage-specific remodeling of the transcriptional and epigenetic landscapes during astrocyte differentiation. Using mouse ESC differentiation into astrocytes as a model system, we identified three transcriptionally and epigenetically distinct stages: (1) a progenitor stage (aNPC), (2) an early stage of astroglial differentiation (eA), and (3) a more advanced stage of astroglial differentiation (lA). We obtained evidence for epigenetic priming along the differentiation axis, indicating that astrogliogenesis follows a dynamic trajectory during which subsequent stages are being orchestrated by preceding ones. By searching for lineage- and stage-specific regulatory elements unique to each stage and inferring their most highly associated TF binding motifs, we were able to pinpoint drivers of the underlying differentiation trajectory: the TFs Nfia, Atf3, and Runx2. These TFs were found to be not only important for expression and maintenance of astroglial marker genes but also critically required for the epigenetic remodeling at the transition between distinct stages. Our molecular analysis of astrogliogenesis supports the notion that differentiation of neural precursors into astrocytes is not a one-step but rather a multi-phase process (Kang et al., 2012). Furthermore, *in vivo* overexpression of these TFs together resulted in an increase in SOX9-positive progenitors at the expense of neurogenesis; however, because of the transient nature of the overexpression, a definitive cell fate could not be tracked. It is possible that the continuous expression of the TFs hampers proper astrocyte development and maturation, and using a system allowing temporally controlled TF expression could further drive the acquisition of an astrocytic phenotype.

A substantial body of prior work has provided compelling evidence for an important role of Nfia at the onset of astrogliogenesis, both in the developing spinal cord and in the cerebral cortex (Cebolla and Vallejo, 2006, Deneen et al., 2006, Kang et al., 2012). However, the precise role of Nfia remains largely elusive. Combined expression of Nfia with Nfib and Sox9 can induce conversion of fibroblasts into astrocyte-like cells (Caiazzo et al., 2015), supporting the notion of an instructive role in astrogliogenesis. The present study showed that Nfia is required for converting primed chromatin into a transcriptionally active one at the transition from aNPC to eA. Nfia also appears to play an important role in suppressing neuronal gene expression. Thus, Nfia may regulate distinct neuronal-fate-suppressing and astrocyte-fate-instructing gene expression modules. The latter module may be co-regulated by Atf3, because there was significant overlap of genes, comprising putative shared direct targets, that were downregulated following knockdown of either TF. Moreover, Nfia became downregulated following Atf3 knockdown, indicating that Atf3 may function upstream of Nfia. Like Nfia, Atf3 was found to be crucial for the conversion of primed to active chromatin. However, in contrast to Nfia, knockdown of Atf3 resulted in the upregulation cell-cycle-related genes, supporting a specific role of Atf3 in cell-cycle exit at the progenitor stage. So far, Atf3 has been largely recognized as a stress response gene (Hai et al., 1999). Stress response such as unfolded protein response (UPR) mediated by Atf4, itself known to induce Atf3, has been shown to regulate the stage-specific balance between direct and indirect neurogenesis from radial glia during cortical development (Laguesse et al., 2015), thus setting a precedent for ATF-mediated cell-fate decisions. Finally, a study identified *Drosophila* Atf3 as a cell polarity response gene. While there is ample evidence for an important role for cell polarity in the regulation of neuronal-fate decisions (Donohoe et al., 2018), virtually nothing is known regarding the acquisition of a glial fate. Our study warrants further investigation into the functional importance of UPR and cell polarity regulation as potential mechanisms in astrogliogenesis.

Virtually nothing is known about Runx2 function in the CNS (Wang and Stifani, 2017). Runx2 plays a key role in osteoblast and chondrocyte differentiation (Komori, 2018). Computational modeling of stem cell-fate decisions predicted Runx2 as a determinant of astrogliogenesis, and Runx2 overexpression induced astroglial differentiation in mouse neural stem cells (Okawa et al., 2016). One particularly intriguing observation was that knockdown of Runx2 resulted in the induction of genes associated with reactive astrocytes, suggesting that Runx2 may be important in repressing a reactive phenotype. Reactive astrogliosis represents in many respects a state of dedifferentiation and involves reacquisition of neural stem cell properties (Robel et al., 2011), which can eventually reactivate neurogenic potential (Magnusson et al., 2014, Nato et al., 2015). It will be interesting to learn whether such dedifferentiation involves downregulation of Runx2.

Consistent with its known function in astrogliogenesis (Bonni et al., 1997, Moon et al., 2002), Stat3 was found to strongly influence astroglial marker expression following knockdown. Surprisingly, however, we failed to observe global changes in H3K27ac at its putative targets. Although we cannot exclude that Stat3 may induce chromatin remodeling via alternative mechanisms, such as sequence-independent recruitment of Stat3, our observation may genuinely indicate that Stat3 acts by transactivating already-accessible targets upon prior chromatin remodeling by pioneer TFs. Given its role in opening chromatin at the transition to the lA stage, Runx2 might be in a prime position to exert such a function. However, activation of Stat3 is also implicated in reactive astrogliosis (Herrmann et al., 2008); hence, Runx2 and Stat3 may act partially antagonistically to each other.

Although our study revealed compelling evidence for dynamic transcriptional and epigenetic remodeling during astrogliogenesis, it also raises intriguing questions regarding the molecular mechanisms that account for the epigenetic writing of specific priming marks at gene loci that subsequently become specifically activated in astrogliogenesis. Furthermore, how the TFs driving astrogliogenesis are activated during this process remains to be investigated. Lastly, we lack molecular insights into how the TFs mediate transition of the chromatin state of the target regulatory elements from primed to active. Deciphering these mechanisms will lead us to a better understanding of how glial and neuronal lineage diverge during development. Our study represents a rich resource for the identification and characterization of further regulators of such key decisions during CNS development.

## STAR★Methods

### Key Resources Table

**Table:** 

REAGENT or RESOURCE	SOURCE	IDENTIFIER
**Antibodies**		

Name	Company	Catalog #
Rabbit polyclonal AQP4	Santa Cruz Biotechnology	sc-20812; RRID:AB_2274338
Rabbit polyclonal ATF3 (C-19)	Santa Cruz	sc-188; RRID:AB_2258513
Rabbit polyclonal ATF3 (EPR19488)	Abcam	ab207434; RRID:AB_2734728
Mouse monoclonal beta III-tubulin	Sigma	T8660; RRID:AB_477590
Rat monoclonal CD44	BD PharMingen	550538; RRID:AB_393732
Rabbit polyclonal Connexin 43	Abcam	ab11370; RRID:AB_297976
Guinea pig polyclonal DCX	Millipore	AB2253; RRID:AB_1586992
Mouse monoclonal FLAG (M2 clone)	Sigma	F1804; RRID:AB_262044
Mouse polyclonal GAPDH	Abcam	ab9484; RRID:AB_307274
Mouse monoclonal GFAP	Sigma	G3893; RRID:AB_477010
Rabbit polyclonal GFAP	Dako	Z0334(29-2); RRID:AB_10013382
Rabbit polyclonal GLAST	Frontier Institute	AB_2571715; RRID:AB_2571715
Rabbit polyclonal H3K4me1	Abcam	ab8895; RRID:AB_306847
Rabbit polyclonal H3K27ac	Abcam	ab4729; RRID:AB_2118291
Rat Monoclonal ASCL1	R&D Systems	MAB2567; RRID:AB_2059503
Mouse polyclonal MYC	Cell Signaling	2276; RRID:AB_331783
Rabbit polyclonal NESTIN	BioLegend	839801; RRID:AB_2565443
Mouse polyclonal NeuN	Millipore	MAB377; RRID:AB_2298772
Rabbit polyclonal Nf-1A (K-21)	Santa Cruz	sc-133816; RRID:AB_10611351
Rabbit polyclonal NFIA	Abcam	ab41851; RRID:AB_944419
Rabbit polyclonal OLIG2	Millipore	AB9610; RRID:AB_570666
Rabbit polyclonal RFP	Biomol	600401379S; RRID:AB_11182807
Rabbit polyclonal Runx2 (M-70) X	Santa Cruz	sc-10758X; RRID:AB_2184247
Rabbit polyclonal S100	Abcam	ab868; RRID:AB_306716
Mouse polyclonal S100-beta	Abcam	ab11178; RRID:AB_297817
Rabbit polyclonal SATB2	Abcam	ab34735; RRID:AB_2301417
Rabbit polyclonal SOX9	Abcam	ab185966; RRID:AB_2728660
HRP anti-Goat IgG	Santa Cruz	SC-2020; RRID:AB_631728
Alexa Fluor 488 goat anti-mouse IgG	Invitrogen	A11001; RRID:AB_2534069
Alexa Fluor 488 donkey anti-mouse IgG	Invitrogen	A21202; RRID:AB_141607
Alexa Fluor 568 goat anti-mouse IgG	Invitrogen	A11004; RRID:AB_141371
Alexa Fluor 633 goat anti-mouse IgG	Invitrogen	A21050; RRID:AB_141431
Alexa Fluor 647 donkey anti-mouse IgG	Invitrogen	A31571; RRID:AB_162542
HRP anti-mouse IgG	Santa Cruz	SC-2005; RRID:AB_631736
Alexa Fluor 488 goat anti-rabbit IgG	Invitrogen	A11008; RRID:AB_143165
Alexa Fluor 488 donkey anti-rabbit IgG	Invitrogen	A21206; RRID:AB_141708
Alexa Fluor 568 goat anti-rabbit IgG	Invitrogen	A11011; RRID:AB_143157
Donkey IgG anti-Rabbit IgG (H+L)-Cy3	Dianova	711-165-152; RRID:AB_2307443
Alexa Fluror 633 goat anti-rabbit IgG	Invitrogen	A21070; RRID:AB_2535731
Alexa Fluor 647 donkey anti-rabbit IgG	Invitrogen	A31573; RRID:AB_2536183
HRP anti-rabbit IgG	Santa Cruz	SC-2004; RRID:AB_631746
Alexa Fluor 633 goat anti-rat IgG	Invitrogen	A21094; RRID:AB_141553
DAPI	Sigma	D9542; Sigma CAS# 28718-90-03

**Bacterial and Virus Strains**		

DH5α Competent Cells	Invitrogen	18265017
One Shot TOP10 Chemically Competent *E. coli*	Invitrogen	C404010

**Chemicals, Peptides, and Recombinant Proteins**		

1 Kb ladder	NEB	N3232S
100 bp ladder	NEB	N3231S
100x Glutamax	Invitrogen	35050-0380
10x Antractic Phos run Buffer	NEB	B0389 S
1-Bromo-3-Chloropropane	Sigma	B9673
1M Hydrochloric acid	Applichem	A1434.1000
2-Mercaptoethanol	Sigma	M3148-100ML
2-Propanol	Sigma	I9516-500ML
Acrylamide	AppliChem	A 1672
Agar	Merck	1119251000
Agarose	Lonza	98200-100
Ammonium persulfat	Amresco	M133-100 g
Ampicillin	Sigma	A5354-10 ml
Ampicillin Sodium Salt	AppliChem	4G017739
Annexin V	BD Biosciences	559934
Antrarctic Phosphase	NEB	M0289S
Aqua-poly mount	Polysciences	18606-20
B27 supplement	Life Technologies	17504-044
BioRad protein assay	GIBCO	500-0006
BrdU	Sigma	B5002-100MG
Bromphenol-blue	Merck	1.08112.0025
BSA(Cell culture)	Sigma	A9418-50 g
Chloroform	Sigma	C2432
Chloroform: Isoamyl alcohol 24:1	Sigma	C0549-1PT
Deoxycholic Acid Sodium salt	Amresco	0613-100G
DEPC water	Roth	K028.2
Dimethylsulfoxide	Sigma	D8418
DMEM	GIBCO	21969-035
DMEM-F12	GIBCO	21331-020
DNase I, RNase free	1000U	EN0525
Donkey serum	Merck Milipore	S30-100ml
dNTP mix	NEB	N0447S
Baytril (Enrofloxacin)	Baytril Bayer	
EGF	PeproTech	AF-100-15-1 mg
EGTA	Sigma	E0396
Ethanol absolute	Sigma	34923
Ethidium bromide	Sigma	46065
Ethylene glycol	Sigma	324558
Ethylenediamine tetraacetic acid Disodiumsalt (EDTA)	Calbiochem	15576-028/324503
FBS	Invitrogen	10270-106
FGF-2	PeproTech	100-18B-100 ug
Formaldehyde	Sigma	F8775-500ML
Gelatin	Sigma	G1890-100 g
Glucose	Sigma	G8270
Glycerol 99%	Sigma	G5516-500ML
Glycine	Merck	8.16013.1000
Glycogen	Roche	10901393001
HEPES acid free	Amresco	0511-250G
HEPES potassium salt	Sigma	H0527
Isoflurane	Isova vet, Centauro	240055
Kanamycin sulfate	Sigma	60615-5 g
L-Glutamax	Invitrogen	35050-0380
LIF	N/A	Self-made
Lipofectamine 2000	Invitrogen	11668-019
Lipofectamine RNAiMAx	Invitrogen	13778-0150
Lithium chloride	VWR	437032G
Magnesium chloride anhydrous	Sigma	M8266
Meloxicam	VITA Laboratories	N/A
Methanol	Sigma	34860-2.5L-R
Milkpowder (skim)	Merck	1.15363.0500
N,N,N’,N’, Tetramethylethylenediamine	Sigma	T9281-50ML
N2 supplement	Life Technologies	17502-048
Natural mouse laminin	Invitrogen	23017-015
Neurobasal medium	GIBCO	21103-049
N-Lauroylsarcosine sodium salt	Sigma	L9150
Non-essential amino acids	GIBCO	1140-035
Nonidet P 40 substitute	Sigma	74385-1L
Oregon Green 488 BAPTA	Life Technologies	O6807
Opti-MEM	GIBCO	31985-047
Paraformaldehyde	Merck	8.18715.1000
PBS pH 7.2 (10x)	500 ml	70013-016
Pen/Strep, 10000u/ml	Invitrogen	15140122
Phenol solution	Sigma	P4557-100 ml
Phenylmethanesulfonyl fluoride	Amresco	0754-5G
Poly-l-ornithine hydrobromide	BD Biosciences	P3655-100 mg
Ponceau-solution	Sigma	P7170-1L
Potassium hydroxide, granular	Merck	1050330500
Propidium iodide	Sigma	P4170-10 mg
Protease inhibitor mix 100x	GE healthcare	80-6501-23
Protein A Agarose beads	Millipore	16-156
Protein G Agarose beads	Millipore	16-266
Proteinase K	Sigma	P2308-25mG
Purple loading dye	NEB	B7024 S
Quick Ligation kit	NEB	M2200S
Rbcl2	Sigma	83980-10 g
Rimadyl Carprofen	Pfizer	N/A
RNase	Applichem	A3832,0050
RNase away	Sigma	83931
Rneasy MiniElute spin columns	QIAGEN	1026497
S2-0.5% Trypsin-EDTA	GIBCO	15400-054
Saponin	VWR	440914Y
SDS-20%	Merck	428018-200ML
SeaBlue Protein Standard	Life Sciences	LC5925
di-Sodium tetraborate Decahydrat	Merck	1.01964.0025
Sodium azide	Sigma	S2202
Sodium bicarbonate	Applichem	A0384
Sodium chloride	Amresco	0241-1KG
Sodium fluoride	Merck	1.06449.0250
Sodium hydroxide	VWR	28244295
Sodium orthovanadate	Alfa Aesor	E11W016
Sodium Pyruvate	Sigma	P2256
Sybr Green	Invitrogen	4367659
T4 DNA Ligase	NEB	M0202S
T4 DNA Ligase Buffer	NEB	B0202S
T4 DNA polymerase	NEB	M0203S
Taq DNA Polymerase	NEB	M0267S
tert.-Butanol	Sigma	308250-1L
Thermo Pol Buffer	NEB	B9004S
Tri reagent	Sigma	T9424-100 ml
Tris Ultrapure	Invitrogen	15504-020
Tris-Hydrochloride	CalBiochem	648317
Triton x 100	Sigma	X100-1L
tRNA	Sigma	R5636-1 ml
Trypan blue	GIBCO	15250-061
Trypsin-EDTA, 0.5%	Life Technologies	15400054
Trypton Soy Agar	Merck	1.05458.0500
Tryptone	Sigma (Fluka)	T9410-250 g
Tween 20	Sigma	P1379-500ML
Vectashield mounting media	Vector	H-1000

**Critical Commercial Assays**		

High Capacity cDNA RT kit	Invitrogen	4368814
Nextera DNA Library Prep Kit	Illumina	FC-121-1030
Power Sybr Green PCR Master Mix	Life Technologies	4367659

**Deposited Data**		

RNA-seq during astrogliogenesis and upon TF depletion	This study	GEO: GSE96539
H3K27ac ChIP-seq during astrogliogenesis and upon TF depletion	This study	GEO: GSE96539
H3K4me1 ChIP-seq during astrogliogenesis	This study	GEO: GSE96539

**Experimental Models: Cell lines**		

ESCs	ATCC	SCRC-1002; RRID:CVCL_5746

**Experimental Models: Organisms/Strains**		

C57BL/6J	The Jackson Laboratory	Jax#000664; RRID:IMSR_JAX:000664

**Oligonucleotides**		

See Table S7 for primer sequences	This study	N/A
See Table S7 for siRNAs sequences	Dharmacon	N/A

**Recombinant DNA**		

pCIDR-dsRed	Pataskar et al., 2016	N/A
pCIDR-Nfia-dsRed	This Study	N/A
pCIDR-Atf3-dsRed	This Study	N/A
pCIDR-Runx2-dsRed	This Study	N/A

**Software and Algorithms**		

ImageJ	NIH	N/A; RRID:SCR_003070
ZEN	Carl Zeiss Microscopy	N/A; RRID:SCR_013672
FACSCanto II using DIVA software	BD Biosciences	N/A; RRID:SCR_001456
HCImage software	Hamamatsu Corporation	N/A; RRID:SCR_015041
Prism	Graphpad	N/A; RRID:SCR_005375
TopHat v.2.0.8	JHU (Trapnell et al., 2009)	N/A; RRID:SCR_013035
SAMTOOLS v0.1.19	University of Birmingham (Li et al., 2009)	N/A; RRID:SCR_002105
HTSeq program v0.5.4p1	Python package (Anders et al., 2015)	N/A; RRID:SCR_005514
DESeq	Bioconductor (Oshlack et al., 2010)	N/A; RRID:SCR_000154
ToppGene	Cincinnati Children’s Hospital Medical Center (Chen et al., 2009)	N/A; RRID:SCR_005726
pheatmap R package	R package	N/A; RRID:SCR_016418
Bowtie v0.12.9	JHU (Langmead, 2010)	N/A; RRID:SCR_016368
QuasR package	Bioconductor (Gaidatzis et al., 2015)	N/A; RRID:SCR_006820
MACS v2.0.10.20120913	Liu’s Lab (Zhang et al., 2008)	N/A; RRID:SCR_013291
ngs.plot.r program	(Shen et al., 2014)	N/A; RRID:SCR_011795
HOMER v4.7	HOMER	N/A; RRID:SCR_010881
findMotifs.pl program	HOMER	N/A; RRID:SCR_016417

### Contact for Reagent and Resource Sharing

Further information and requests for resource/reagents should be directed to and will be fulfilled by the Lead Contact, Prof. Dr. Benedikt Berninger (benedikt.berninger@kcl.ac.uk).

### Experimental Model and Subject Details

The handling of the mice and all experimental procedures were conducted in accordance with the European Union guidelines on the use and welfare of experimental animals (2010/63/EU). Experimental procedures were approved by the State of Rheinland Pfalz, Germany (23177-07/G17-1-067) or Spanish Ministry of Agriculture (RD 1201/2005 and L 32/2007) and the Cajal Institute, CSIC Animal Experimentation Ethics Committees and the Community of Madrid (Ref. PROEX 223/16). Animals were housed on a 12:12 hr light-dark cycle, with free access to food and water. None of the mice used were involved in previous procedure or drug treatment. Both male and female embryos were used and randomly allocated to experimental groups.

#### Cell culture

mESCs derived from C57/BL-6 mice were cultured in DMEM supplemented with 10% FBS, 2 mM Glutamax, 2 mM sodium pyruvate, 2 mM non-essential amino acids, Leukemia-inhibitory factor (LIF) and 50 μM β-mercaptoethanol. The cell medium was changed every day, and the cells were passaged every second day using 0.05% trypsin and plated on 0.2% gelatin coated plates. Using the protocol proposed by Conti et al., the ESCs were further differentiated into aNPC (Conti et al., 2005, Pollard et al., 2006). Briefly, the ESCs were passaged and grown in N2/B27 media containing 0.5x DMEM-F12, 0.5x N2 supplement, 0.5x B27 supplement, 0.5x Neurobasal media, 2 mM Glutamax and 2 mM Penicillin-Streptomycin for 7 days on 0.2% gelatin coated plates. The cells were trypsinized and grown on uncoated dishes for the following 7 days in NSA media containing 10 ng/ml FGF2 and 10 ng/ml EGF to form cell aggregates. The formed aggregates were further passaged and grown on 0.2% Gelatin coated plated to obtain the monolayer of the aNPC. To generate the astrocytes, the aNPC were differentiated in differentiation media containing DMEM, 1% FBS, 1x B27, LIF, 2 mM Glutamax and 2 mM Penicillin-Streptomycin for 1, 5 and 21 days. For differentiation of aNPC into neurons, cells were kept in neurobasal media, along with B27 for 2 weeks. 293T cells were cultured in DMEM, 10% FBS, Penicillin-Streptomycin and Glutamax.

#### Primary astrocyte culture

The primary astrocyte culture was prepared from the brains of P5-P7 C57/BL6 mice using a previously described protocol (Heinrich et al., 2011). Briefly, the cortices were dissected to remove the white matter and meninges and cultured in DMEM-F12 media supplemented with 1x B27, 10% FBS, 2 mM Glutamax and 2 mM Penicillin-Streptomycin.

### Method Details

#### Immunofluorescence and Brdu labeling

The cells were grown on coverslips, fixed with 4% paraformaldehyde and permeabilized with 0.2% Triton X-100 for 5 minutes at room temperature. Subsequently, the cells were blocked with 5% BSA for 30 minutes, incubated with primary antibodies (mouse anti-GFAP-1:500, G3893, Sigma; rabbit anti-GFAP-1:200, Z0334(29-2), DAKO; rabbit anti-AQP4-1:200, sc-20812, Santa Cruz; mouse anti-S100B, ab11178, Abcam; rabbit anti-GLAST-1:200, AB_2571715, Frontier Institute; rabbit anti-CX43-1:200, ab11370, Abcam; rabbit anti-OLIG2-1:200, AB9610, Milipore; rat anti-ASCL1-1:200, MAB256, R&D systems; rabbit anti-NES-1:200, 839801, BioLegend; anti-guinea pig anti-DCX-1:200, AB2253, Milipore; rabbit anti-SOX9-1:200, ab185966, Abcam and rat anti-CD44-1:200, 550538, BD PharMingen) for 1 hour, and then incubated with a fluorochrome-labeled secondary antibody for 45 minutes at room temperature. The coverslips were counterstained with DAPI and imaged under a confocal laser-scanning microscope. The data were processed using Adobe Photoshop software. For the Brdu labeling, the aNPC were differentiated into astrocytes for 1 and 5 days, labeled with 30 μM Brdu for 8 hours. Cells were fixed in 70% ethanol and denatured by using 2N HCL/0.5% Triton X-100 for 30 minutes. HCL was neutralized by 0.1 M Na2B4O7 pH 8.5 buffer for 2 minutes. Cells were washed with 0.5%Tween-20/1% BSA/PBS buffer before proceeding for Brdu staining (1:200, B5002, Sigma) as above.

#### Immunoblotting

Immunoblotting was performed as described previously (Tiwari et al., 2013) using following antibodies: mouse anti-GFAP (1:1000, G3893, Sigma), rabbit anti-CX43 (1:000, ab11370, Abcam), rat anti-ASCL1 (1:1000, MAB2567, R&D systems), rabbit anti-NES (1:1000, 839801, BioLegend), rabbit anti-OLIG2 (1:1000, AB9610, Milipore), rabbit anti-NFIA (1:1000, ab41851, Abcam), rabbit anti-RUNX2 (1:1000, sc-10758 X, Santa Cruz), rabbit anti-GAPDH (1:1000, ab9484, Abcam) and mouse anti-FLAG (1:1000, F1804, Sigma). Briefly, cells were lysed using RIPA buffer (50 mM HEPES pH 7.5, 150 mM NaCl, 5 mM EGTA, 1.5 mM MgCl2, 1% Glycerol, 1% Triton X-100) and protein concentration was measured using Biorad reagent (Life Technologies) following manufacturer’s protocol. 50 μg lysate was mixed with final 1x Laemelli buffer (5x Laemelli buffer: 10% SDS, 50% glycerol, 25% 2-mercaptoethanol, 0.02% bromphenol blue and 0.3125 M Tris HCl, pH approx. 6.8.), then run at 150 V and transferred at 100 V for 2-hours with BioRad Mini-PROTEAN and Mini Trans-Blot Cell tanks respectively. Subsequently, the membranes were blocked with 5% non-fat dry milk in TBST for 1 h at room temperature and incubated in primary antibodies diluted in 5% non-fat dry milk in TBST for overnight at 4°C. The membranes were washed three times for 5 minutes with TBST and incubated in horseradish peroxidase coupled secondary antibody (in 5% non-fat dry milk) for 1 hour at room temperature. After three 5 minutes washes with TBST, membranes were incubated with ECL solution (Amersham ECLTM Prime Western Blotting Detection Reagent) according to the manufacturer’s instructions and imaged with the Peqlab Fusion-SL system using the fusion software version 15.16 by Vilber Lourmat.

#### Ca^2+^ Imaging

eA and lA were incubated with 10 μM OGB1, diluted in culture medium, for 45 min at 37°C in a humidified atmosphere. After the incubation time, cells were washed with warm culture media and analyzed within 2 hr (Lee et al., 2015). Cells were placed in a recording chamber mounted on a fluorescent microscope (Zeiss Axio Imager A2) and superfused with artificial cerebrospinal fluid (ACSF) (in mM): NaCl, 125; KCl, 2.5; NaHCO_3_, 25; NaH2PO_4_, 1.25; CaCl2, 2; MgCl_2_, 1 and glucose, 25, saturated with 5% CO_2_ and 95% O_2_ (pH 7.4, 30-32°C). Cells were visualized with a 40x (0.7 NA) objective and movies (15 s) were acquired with a frequency of 8.9 Hz with a Hamamatsu Orca-0.3G camera and HCImage software (Lee et al., 2015). During acquisition, we tested the generation of Ca^2+^ waves in astrocytes using mechanical stimuli with a glass pipette which was placed right above the membrane of cultured astrocyte (resistance of 5-10 MΩ, which corresponds to a tip opening of 1-2 mm) (Lee et al., 2015). For quantification, we defined regions of interest (ROIs) in cells surrounding the site of mechanical stimulus. Intensity measurements for ROIs are expressed as the change of fluorescence relative to background fluorescence (ΔF/F0) and were calculated with ImageJ (ROI manager) (Lee et al., 2015).

#### siRNA transfection and plasmid transfection

For the siRNA (Dharamcon) transfection, Lipofectamine RNAiMax (Invitrogen) was used according to manufacturer’s instructions to knockdown the candidate TFs. The candidates were first knocked down in aNPC for −2 days to reduce the basal level expression and were then re-transfected with siRNA at day 0 and kept in aNPC media for 1 day and changed to astromedia next day for 1 day experiment (Nfia and Atf3). On the other hand, for the 5 days experiment (Runx2), day 2 astrocytes were re-transfected with siRNA in astrocyte differentiation media. 293T cells were transfected with pCIDR-dsRed or pCIDR-TF-dsRed plamids by using Lipofectamine 2000, according to manufacturer’s instructions.

#### RNA purification, cDNA preparation and Quantitative RT-PCR

RNA was extracted using the Tri-reagent from sigma, and cDNA was prepared according to the manufacturer’s instructions (Applied Biosystems) using 1 μg of initial RNA material. The cDNA was used to quantify the gene expression on a StepOne plus real-time PCR machine. The primer sequences are provided in Table S7.

#### Chromatin Immunoprecipitation

The ChIP experiments were performed as previously described (Tiwari et al., 2013). In brief, crosslinked chromatin was sonicated to achieve an average fragment size of 500 bp. Starting with 60 μg of chromatin and 5 μg of the anti-H3K27ac (Abcam), anti-Nfia (Santa Cruz), anti-Atf3 (Santa Cruz) and anti-Runx2 antibodies (Santa Cruz), the samples were immunoprecipitated and then de-crosslinked to release the DNA fragments. In total, 1 μL of the ChIP material and 1 μL of the input material were used for the quantitative real-time PCR using specific primers covering the motifs of Nfia, Atf3 and Runx2 in eA and lA. Primers covering an intergenic region were used as a control. The efficiencies of the PCR amplification were normalized to the PCR product of the intergenic region. Primer sequences provided in Table S7.

#### Transcriptome analysis

The transcriptome samples under the astroglial differentiation (aNPC, eA and lA), control and TF knockdown conditions (GSE96539) were derived in triplicate for astroglial differentiation and in biological replicates for depletion studies. The RNaseq output in FASTQ format was subjected to an initial quality assessment using FASTQC v2.6.14. After the quality check, the files were used for the read alignment to the mouse genome (mm9) with UCSC annotations (Karolchik et al., 2014) using TopHat v.2.0.8 (Trapnell et al., 2009); then, the uniquely mapped reads were retained in the output BAM file. SAMTOOLS v0.1.19 (Li et al., 2009) was used for the file format conversions and sorting of the alignment file. The raw read counts per gene were calculated using HTSeq program v0.5.4p1 (Anders et al., 2015), and the read count data matrix was then subjected to library-size normalization and differential expression analyses using DESeq (Oshlack et al., 2010). An FDR cutoff of 0.1 was used to call the differentially expressed genes. The transcriptomes from the *in vitro* astroglial differentiation were normalized together, while those from the control and TF KD were normalized independently. The GO term analysis of all gene lists (Gene Symbol) was performed using ToppGene (Chen et al., 2009). Heatmaps depicting the gene expression were plotted using pheatmap R package. The analyzed microarray data from GSE9655 (Cahoy et al., 2008) were directly downloaded from GEO, while the RNaseq data from GSE73721 (Zhang et al., 2016) were analyzed as described above and compared to the RNaseq data using a linear regression analysis. Gene lists derived from the RNaseq analysis are supplied in Table S1.

#### ChIP-seq and motif analysis

The ChIP-sequencing output in FASTQ format was subjected to a quality check using FASTQC v2.6.14 (Andrews). ChIP-seq was performed to detect the histone markers H3K27ac and H3K4me1 during each stage of astroglial differentiation (GSE96539) and H3K27ac under the control and shortlisted TF knockdown conditions. Bowtie v0.12.9 (Langmead, 2010) was used to align the reads uniquely, i.e., each read was maximally aligned to one position, to mm9 genome with UCSC annotations (Karolchik et al., 2014). The alignment output files from two biological replicates were merged together after checking for correlations across the replicates using the SAMTOOLS v0.1.19 (Li et al., 2009) merge function. SAMTOOLS v0.1.19 (Li et al., 2009) was used for the alignment file format conversions and sorting of alignment output files. The WIGGLE files for the alignment files were generated using QuasR package (Gaidatzis et al., 2015). The peaks were computed without providing input with MACS v2.0.10.20120913 (Zhang et al., 2008) using the default parameters. The enrichment was calculated using formula as previously described (Pataskar et al., 2016). The heatmaps and the density plots representing the ChIP-seq normalized read distribution over the shortened genomic region were plotted using the ngs.plot.r program (Shen et al., 2014).

HOMER v4.7 was used to obtain annotation information for the peaks, including the distance to the nearest TSS and the nearest gene. The peaks were associated with their nearest genes if they were either intergenic and less than 50KB from the TSS or at promoters, intron and exons.

A motif analysis was performed by HOMER v4.7 using a GC% normalized background control on the shortlisted genome coordinate sets using the findMotifs.pl program. Motif scanning of the complete genome was performed using the scanMotifsGenome.pl program in HOMER v4.7.

#### ATAC

ATAC library preparation was performed exactly as described (Buenrostro et al., 2013). Briefly, 50,000 eA and lA cells were incubated with 0.1% NP-40 to isolate nuclei. Nuclei were then transposed for 30 min at 37°C with adaptor-loaded Nextera Tn5 (Illumina, Fc-121-1030). Transposed fragments were directly PCR amplified and used for qRT-PCR.

#### Cell growth assay

For growth curves, cells were seeded in each well of a 24-well plate and cell numbers were assessed over the time periods indicated by using a Neubauer counting chamber upon TF depletion. For cell cycle analysis, cells were trypsinized and fixed overnight in 70% ice-cold ethanol, washed twice with PBS, and resuspended in sodium citrate buffer with 5 μg/ml PI overnight. Stained cells were analyzed on a FACSCanto II using DIVA software.

#### Apoptosis assay

Apoptosis assays were performed using a Cy5 Annexin V antibody according to the manufacturer’s instructions (BD Biosciences). Stained cells were filtered with a 40μm mesh and analyzed on a FACSCanto II flow cytometer using DIVA software.

#### In utero electroporation

E15.5 pregnant C57BL/6 mice were anesthetized with 1.5%–2.8% isoflurane (Isova vet, Centauro) in pure O_2_ and received an injection of enrofloxacin (5 mg/kg, s.c.; Baytril Bayer) and meloxicam (300 μg /kg, s.c.; VITA Laboratories)/ Rimadyl (carprofen; 5 mg/kg; Pfizer) before starting the surgical procedure. The surgical area was constantly maintained moistened with a physiological saline solution. The abdominal cavity was opened, and the uterine horns were carefully extracted. For each embryo, 1 μL of DNA solution was injected into the lateral ventricle using a glass capillary, and 5 consecutive electric pulses of 37 V (50 ms each, 950 ms intervals) were delivered through platinum electrodes (3 mm diameter) using a BTX electroporator (Holliston). The uterine horns were then replaced in the abdominal cavity, and the abdomen was sutured. The DNA consisted of a pCAG-IRES-dsRedexpress (pCIDRE; 4 μg) (Pataskar et al., 2016) plasmid as control, or encoding NFIA, ATF3 or Runx2.

#### Immunohistochemistry and image analysis

For the in utero electroporated brains analyzed at E18.5, the pregnant mice were sacrificed, and the embryos were extracted from the uterine horns. For analysis at postnatal stages, animals were perfused with a saline solution (NaCl 0,9% in PB 0,1M) followed by 4% paraformaldehyde (PFA). Harvested embryonic brains were placed in 4% PFA for 48 h for fixation and postnatal brains were post-fixed in 4% PFA for 24 h. The brains were embedded in agarose (3%, in PBS) and cut into 50 or 70 μm (for postnatal and embryonic brains, respectively) serial coronal sections using a vibrating microtome (Microm HM650V, Thermo Scientific). The sections were then stored in a cryoprotective solution (20% glucose, 40% ethylene glycol, 0.025% sodium azide, and 0.05 M phosphate buffer, pH 7.4). For immunohistochemistry, free-floating sections were incubated for 90 min at room temperature (RT) in a blocking solution (5% Donkey serum, 0.3% Triton X-100 in TBS 0.1 M, pH 7.6). The primary antibodies, which were diluted in blocking solution, were applied for 2 hours at RT and overnight at 4°C. The following antibodies were used: donkey anti-RFP (1:500, 600401379S, Biomol) to detect the pCIDRE-electroporated cells, rabbit anti-SOX9 (1:200, ab185966, Abcam), rabbit anti-SATB2 (1:200, ab34735, Abcam), mouse anti-TUBB3 (1:500, T8660, Sigma), rabbit anti-GLAST (1:300, AB_2571715, Frontier Institute), mouse anti-MYC tag (1:200, 2276S, Cell signaling), and mouse anti-NeuN (1:500, MAB377, Milipore). The appropriate secondary antibodies were diluted in blocking solution and applied for 1 hour at room temperature. The sections were then counterstained with DAPI and mounted with Vectashield (Vector Laboratories) mounting medium. Image stacks were acquired under an SP5 confocal microscope (Leica) with a 20x dry objective (NA 0.7) or a 40x oil objective (NA 1.3). For Figure 7M, tilescans showing the dispersion pattern of RFP +ve cells were acquired under an SP5 confocal microscope (Leica) with a 40x oil objective (NA 1.3). Output 2D images were generated in ImageJ using the Maximum Intensity Projection (MIP) method. The quantification of the distribution of the electroporated cells was performed in 3 animals/group (n = 3) with 2 sections/brain.

### Quantification and Statistical Analysis

Statistical significance was evaluated by One-Way ANOVA, followed by Dunnett’s test for multiple group comparisons or by t test for 2-groups comparison, using the SPSS Statistics 22 software for Figures 7, S1, S5, and S7. P values represent ^∗^ < 0.05; ^∗∗^ < 0.01; ^∗∗∗^ < 0.001. The data are expressed as the mean ± SD. The number of experiments and biological samples used is specified in figure legends and Star Methods. The bar plots were generated using Prism software.

### Data and Software Availability

All relevant data are available from authors. All the software used in this work are described in the relevant Star Methods sections. RNA-seq and ChIP-seq data have been deposited (GEO: GSE96539).

## Supplemental Information

Document S1. Figures S1–S7

Table S1. Differentially Expressed Genes following Depletion of Nfia, Atf3 in eA and Runx2 in lA as well as between and within Various Stages of Astrogliogenesis versus Neurogenesis *In Vitro*, Related to Figures 1, 3, and 5

Table S2. Differentially Expressed Genes (Log2 Fold Change) in Each Cluster Shown in Figure 1C as well as Their Gene Ontologies, Related to Figure 1

Table S3. H3K27ac Enrichment during Astrogliogenesis and Neurogenesis and H3K4m1 Enrichment during Astrogliogenesis on Genomic Loci of Different Clusters Shown in Figure 2J, Related to Figure 2

Table S4. Comparison of TF Binding Motifs Enriched at H3K27ac Sites during Astrogliogenesis and Neurogenesis, Related to Figure 3

Table S5. Genes Commonly Regulated following Nfia and Atf3 Depletion and Having H3K27ac Enrichment at their Regulatory Elements, Related to Figure 6

Table S6. Percentage of Distribution of RFP +ve (E18.5 and P5) as well as SOX9 (E18.5 and P5), SATB2 (E18.5), and NeuN (P5) +ve Cells among RFP +ve Cells upon *In Utero* Electroporation of pCIDRE or pCIDRE-TF(s) in E15.5 Cortical Progenitors, Related to Figure 7

Table S7. Sequences of Primers Used for ChIP-qPCRs and qRT-PCRs, as well as siRNAs Used in This Study, Related to Figures 1, 2, 3, 5, 6, and 7

Document S2. Article plus Supplemental Information
