# Supplementary material for: Stage-Specific Transcription Factors Drive Astrogliogenesis by Remodeling Gene Regulatory Landscapes
Source: Cell Stem Cell. 2018 Oct 4;23(4):557–571.e8. doi: 10.1016/j.stem.2018.09.008 (PMC6179960; doi:10.1016/j.stem.2018.09.008)
Supplement: Document S1. Figures S1–S7 [file mmc1.pdf]

Cell Stem Cell, Volume *23*

Supplemental Information

Stage-Specific Transcription Factors Drive Astrogliogenesis by Remodeling Gene Regulatory Landscapes

Neha Tiwari, Abhijeet Pataskar, Sophie Péron, Sudhir Thakurela, Sanjeeb Kumar Sahu, María Figueres-Oñate, Nicolás Marichal, Laura López-Mascaraque, Vijay K. Tiwari, and Benedikt Berninger

Supplemental Figure 1 (Related to Figure 1)

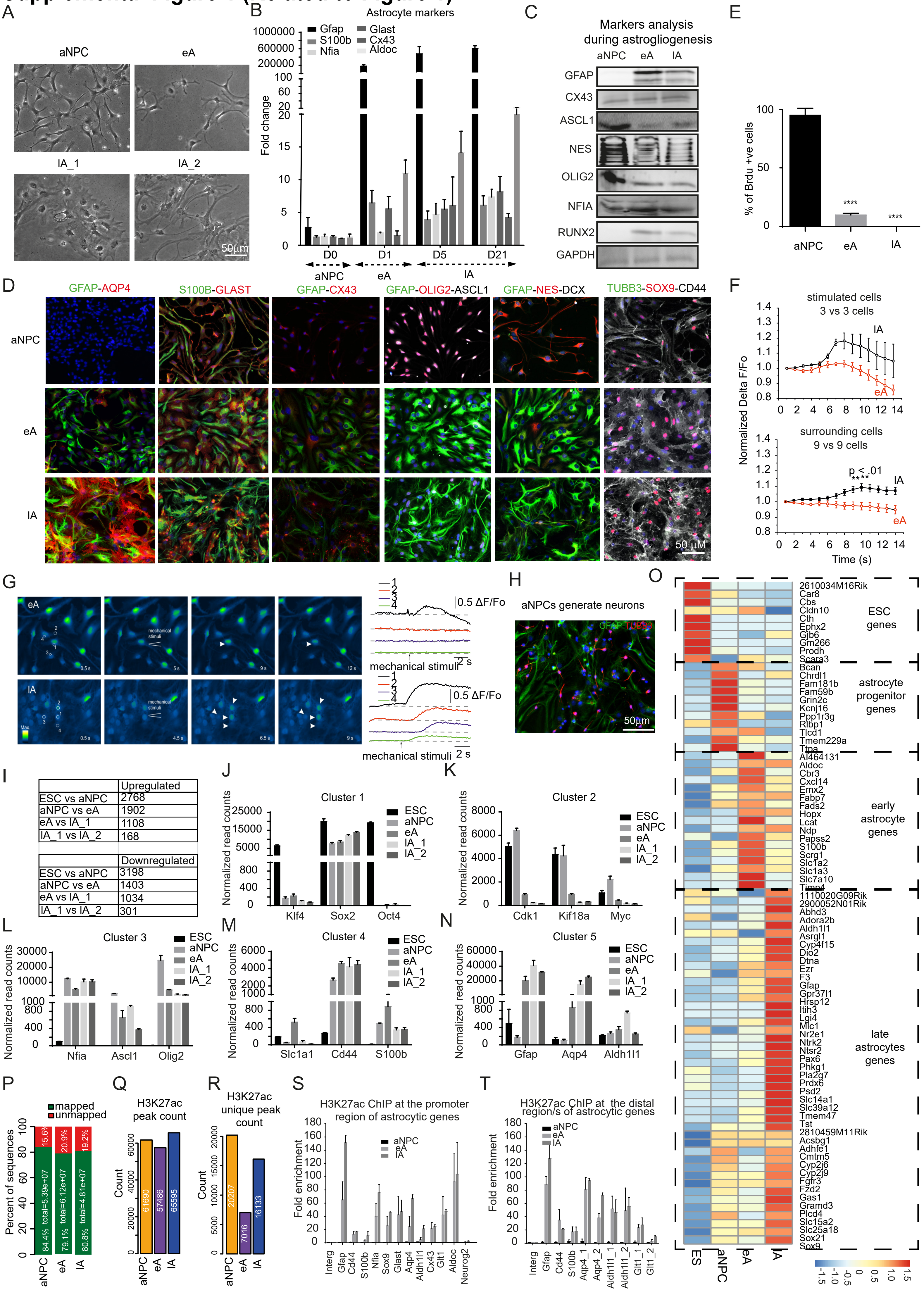

Supplemental Figure 2 (Related to Figure 2)

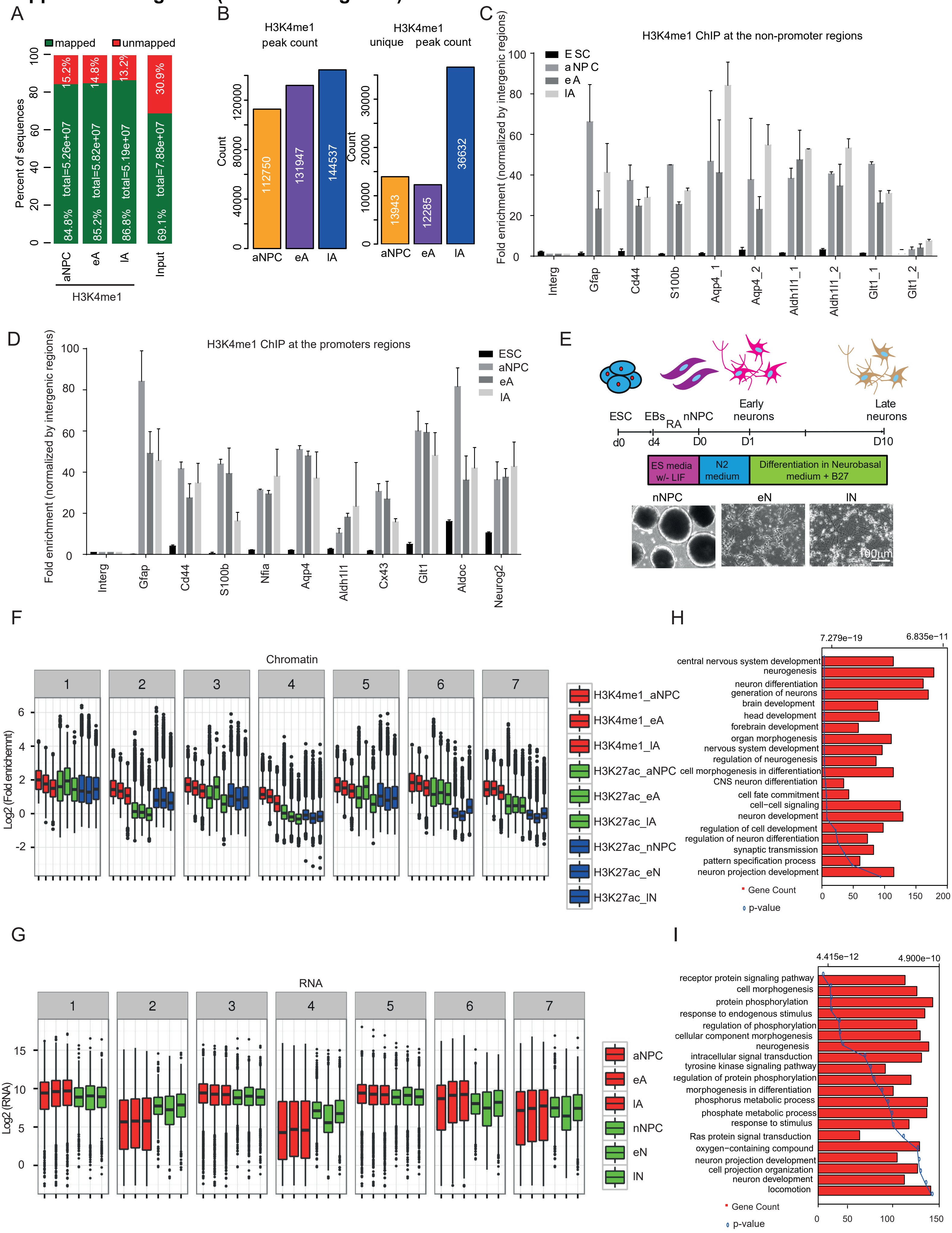

Supplemental Figure 3 (Related to Figure 3)

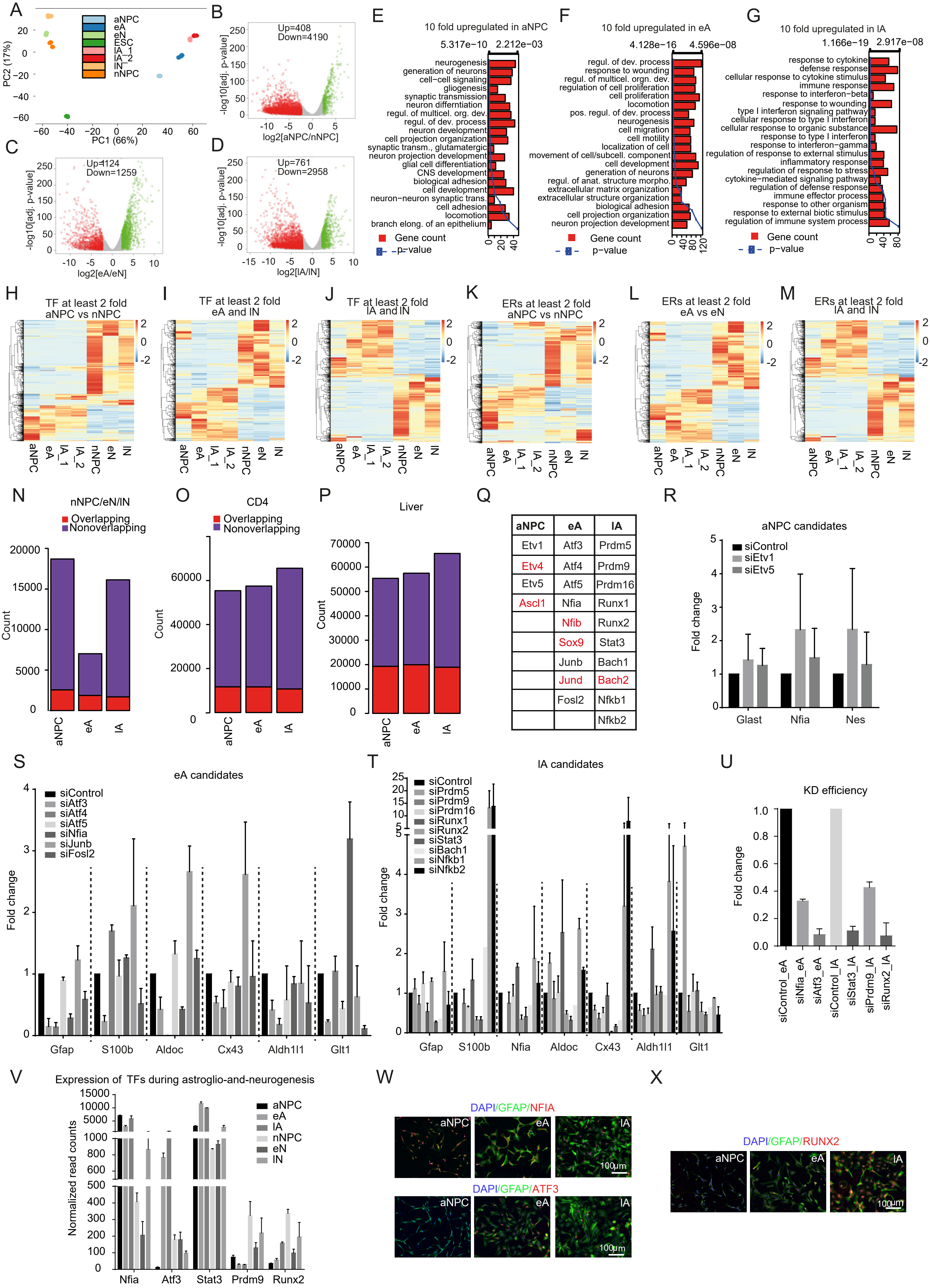

Supplemental Figure 4 (Related to Figure 4)

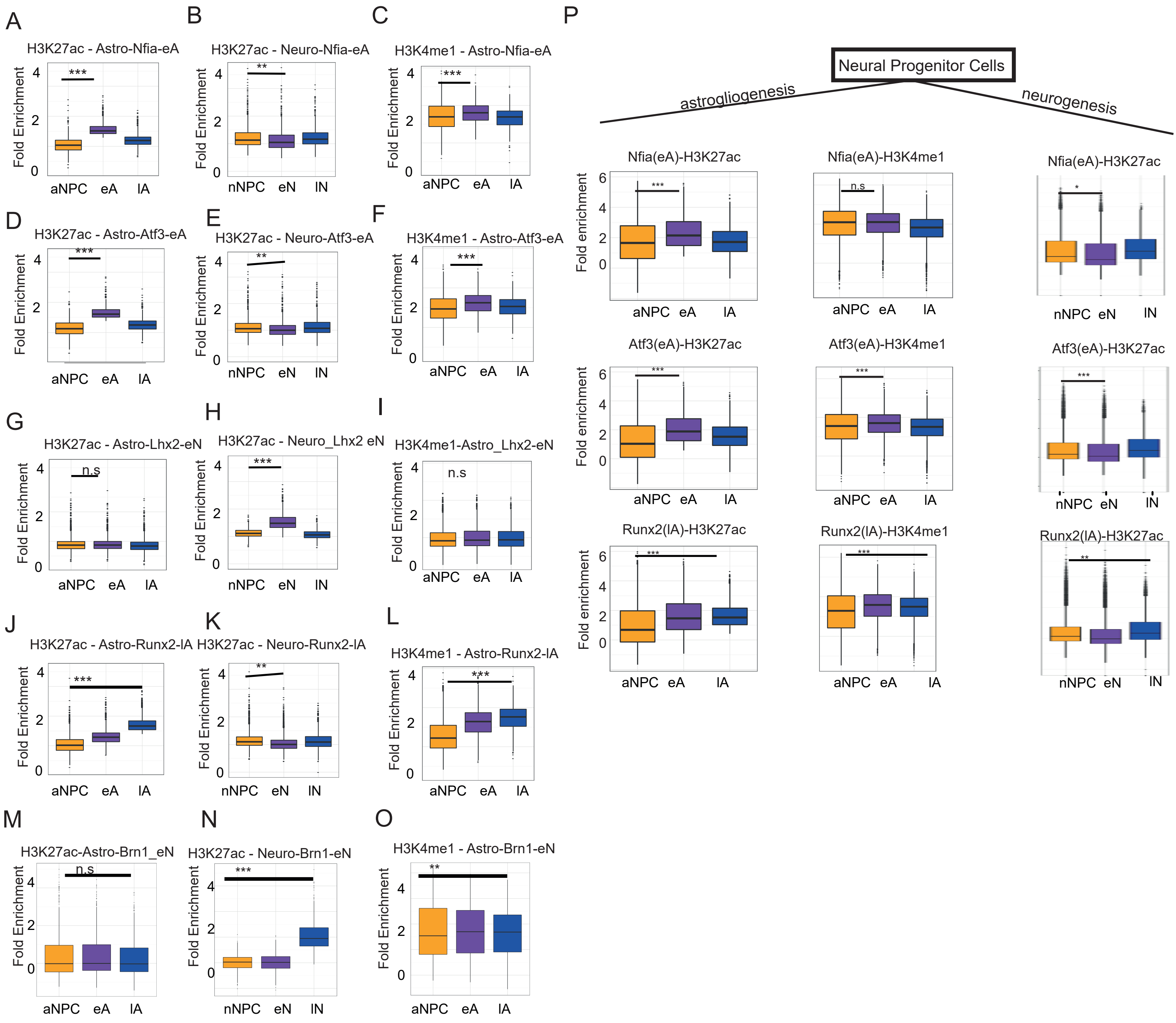

Supplemental Figure 5 (Related to Figure 5)

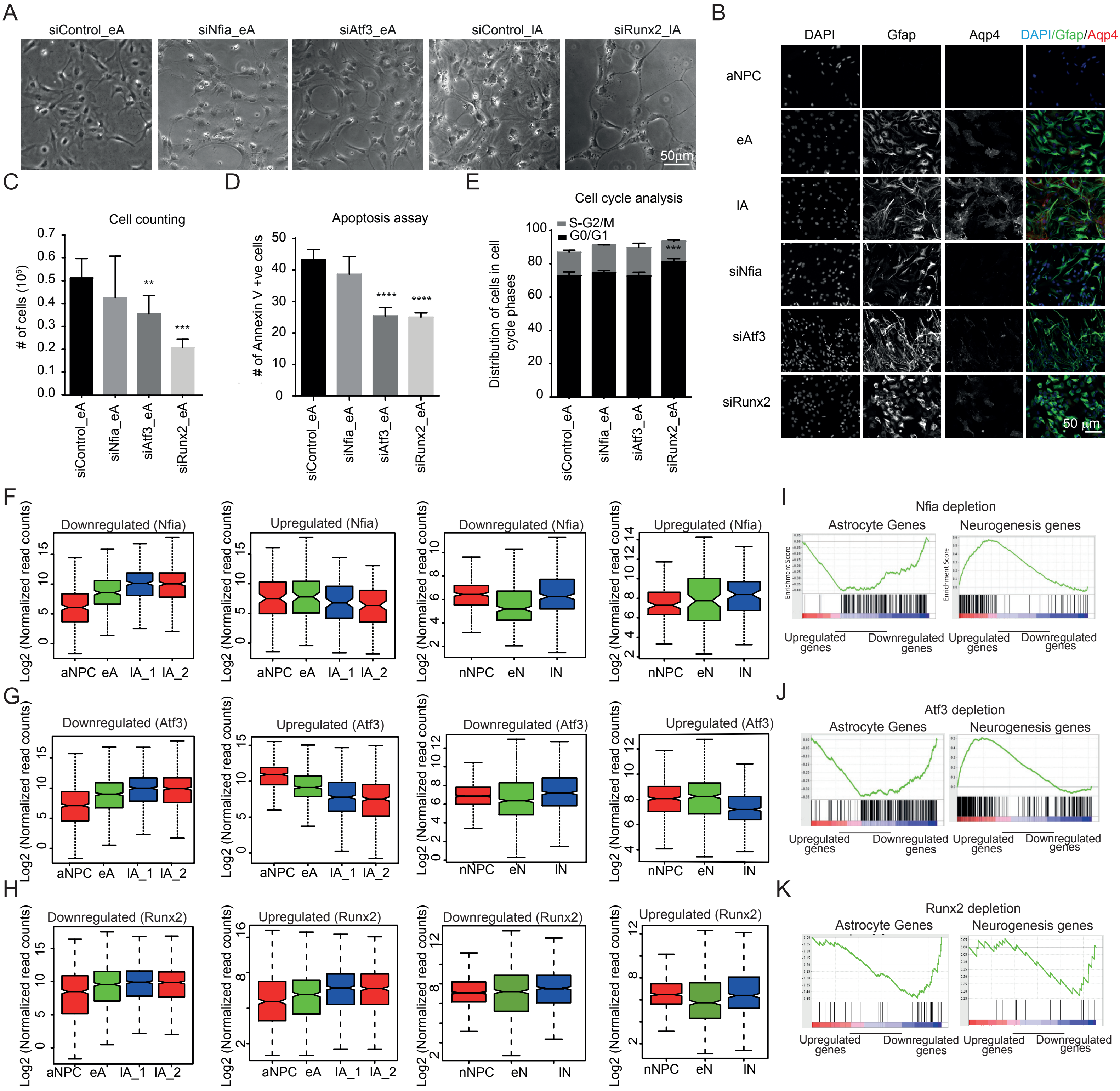

Supplemental Figure 6 (Related to Figure 6)

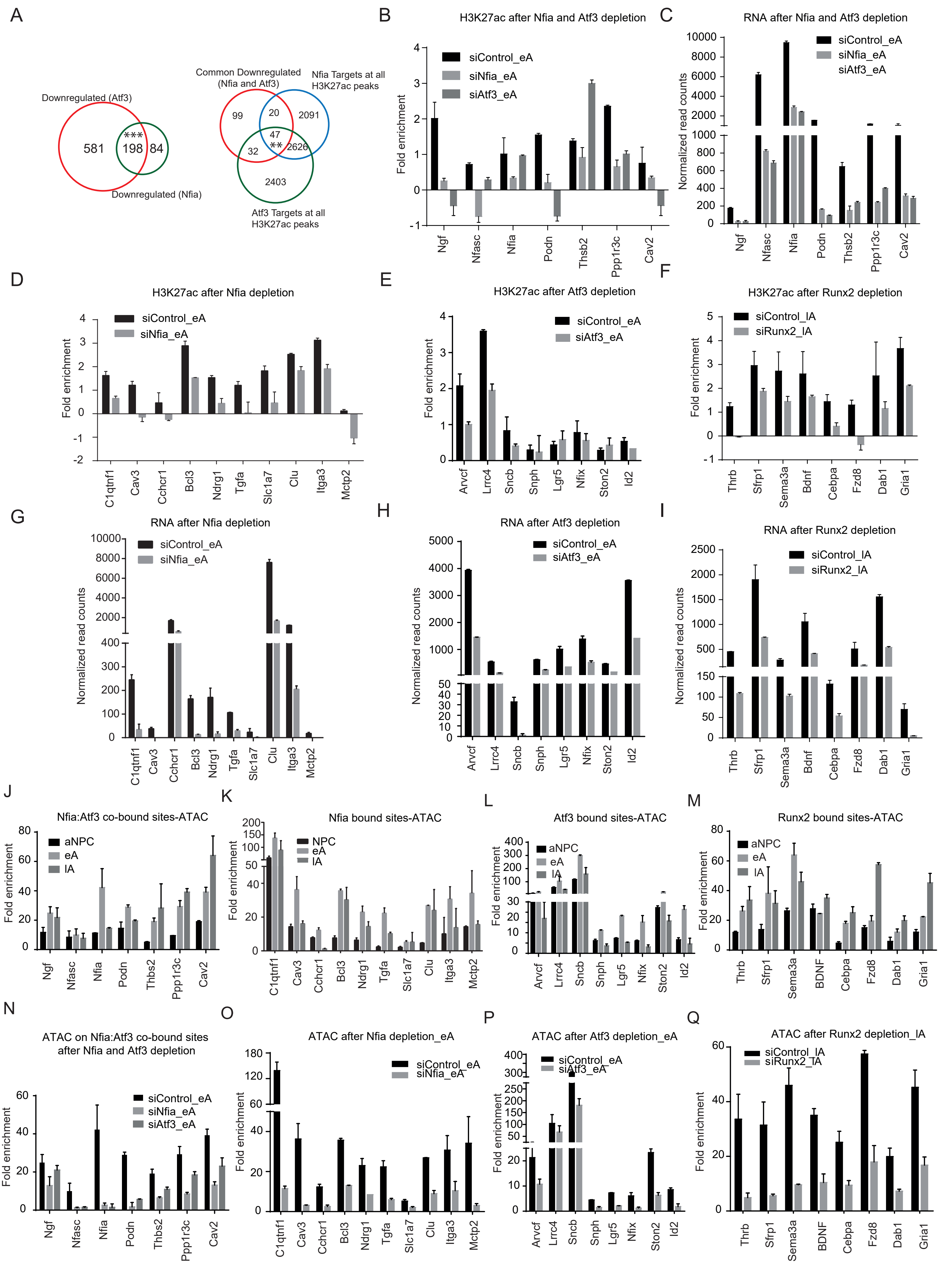

Supplemental Figure 7 (Related to Figure 7)

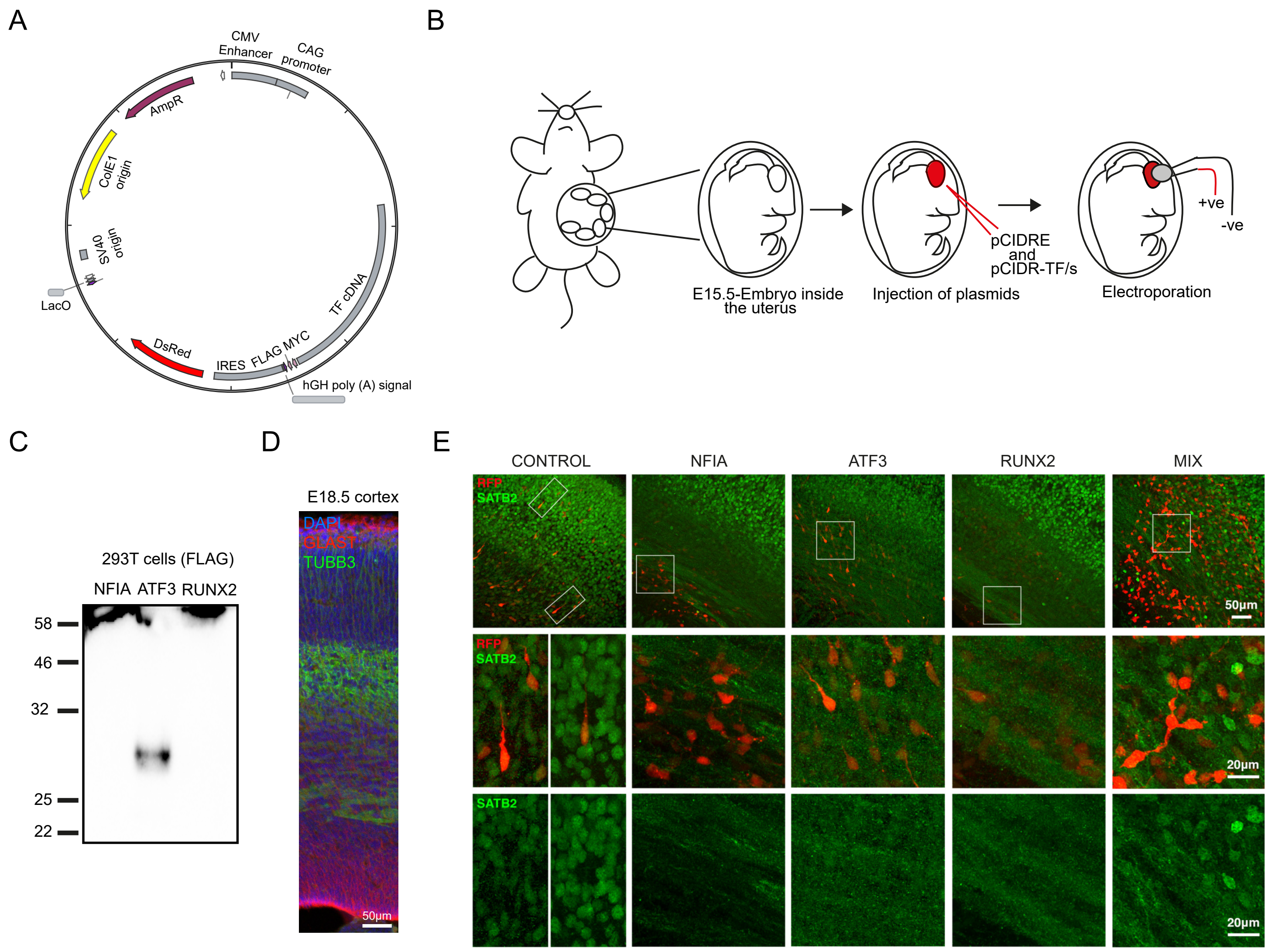

SUPPLEMENTAL INFORMATION

Figure S1 (Related to Figure 1):

(A) Bright-field images showing the morphology of ESC-derived astrocytes during different differentiation stages (aNPC, eA, lA_1 and lA_2). Scale bar is 50 µm. (B) qPCR depicting the expression, represented as fold change, of astroglial markers (Gfap, S100b, Nfia, Glast, Cx43, and Aldoc) in aNPC, eA, lA_1 and lA_2. (C) Western blot analysis to show the expression of astroglial markers (GFAP, CX43), progenitor markers (NES, OLIG2, NFIA) and Runx2 during astrogliogenesis. (D) Immunofluorescence staining of various astrocyte (GFAP, AQP4, S100B, GLAST, CX43, SOX9, CD44), progenitor (OLIG2, ASCL1, NES) and neuronal (TUBB3) markers in the ESC-derived astrocytes during different phases of astrogliogenesis, including the aNPC, eA and lA. The scale bar is 50 µm. (E) Brdu +ve cells were quantified by incorporating Brdu in aNPC, eA and lA for 8 hours. (F) Propagation of calcium waves upon mechanical stimulation of eA and lA membrane in stimulated (top) and surrounding (bottom) cells. (G) Pictures depicting the propagation of calcium waves (arrowheads) in stimulated and surrounding cells upon mechanical stimulation in eA (upper panels) and lA (lower panels). $\Delta F/F0$ analysis of ROIs (open circles) showed the time course of [Ca_{+2}]i increase after mechanical stimuli application. (H) Immunofluorescence staining for GFAP and TUBB3 in aNPC cells kept in differentiation media for 2 weeks (I) Table depicting the quantification of the significantly differentially expressed genes in respective comparisons. (J-N) Normalized read counts showing the expression of genes in each cluster depicted in Figure 1C (O) Heat map visualizing the expression of commonly regulated genes in the glial network and our dataset during astrogliogenesis (P) Quality control plot depicting the number of H3K27ac reads aligned per sample compared to the total sequenced reads after merging the two biological replicates. (Q) Bar plot depicting the H3K27ac peak counts in aNPC, eA and lA. (R) Bar plots showing the number of H3K27ac peaks unique to a particular cell type, i.e., either aNPC, eA or lA. (S, T) ChIP-qPCR to analyze the enrichment of H3K27ac in the promoter (S) and distal regulatory region (T) of astrogliogenic genes in aNPC, eA and lA.

Figure S2 (Related to Figure 2):

(A) Bar plot depicting the number of H3K4me1 reads aligned per sample compared to the total sequenced reads after merging the two biological replicates. (B) Left side: Bar plot depicting the H3K4me1 peak counts in aNPC, eA and lA. Right side: Bar plots showing the H3K4me1 peaks unique to a particular cell type, i.e., aNPC, eA or lA. (C, D) Bar graphs representing the ChIP-qPCR validation of the non-promoter (C) and promoter (D) regulatory regions of representative

astroglial and neuronal marker genes. (E) Schematic representation of neuronal differentiation from ES cells. Scale bar is 100 µm. (F) Box plots depicting the ChIP-seq enrichment of H3K27ac during astrogliogenesis (green) and neurogenesis (red) and H3K4me1 enrichment during astrogliogenesis at genomic regions differentiated by the clusters shown in Figure 2J. (G) Same as (F) but a depiction of the expression of genes during astrogliogenesis (red) and neurogenesis (green) that are associated with genomic regions that are differentiated by the clusters shown in Figure 2J. (H, I) Bar and line plots depicting the top gene ontologies enriched with genes associated with the regions depicted in cluster 2 (H) and clusters 6 and 7 (I) in Figure 2J. Main x-axis shows gene count while alternate x-axis on top shows p-values.

Figure S3 (Related to Figure 3):

(A) Principal component analysis plot depicting the distribution of the transcriptomes in ESC, aNPC, eA, lA_1, lA_2, nNPC (neural progenitors), eN (early neurons) and lN (late neurons). (B-D) Volcano plot showing the differentially expressed genes in the comparison of the transcriptomes (Red: downregulated, Green: upregulated; Grey: Not changing) between aNPC and nNPC (B), eA and eN (C) and lA and lN (D). (E-G) Top enriched gene ontologies for genes that are at least 10 fold or more expressed in aNPC compared to nNPC (E), eA compared to eN (F) and lA compared to lN (G) are represented as bar and line plots. (H-J) Heatmap depicting the expression of TFs that are at least 2 fold or more expressed in aNPC compared to nNPC (H), eA compared to eN (I) and lA as compared to lN (J). (K-M) Same as (H-J) but for the expression of epigenetic regulators. (N-P) Overlap of H3K27ac enriched sites during astrogliogenesis with neurogenesis (nNPC, eN and lN; N), CD4 (O) and liver (P)'s H3K27ac sites. (Q) Selected candidate TFs for each stage of astrogliogenesis. The one highlighted with red color did not give significant depletion in their expression upon siRNA treatment. Thus, they were excluded from the studies. (R-T) siRNA depletion of stage-specific selected candidates in their respective stages and analysis of progenitor and astroglial marker expression by q-PCR. (U) qPCR following the Nfia and Atf3 depletion in eA and the Stat3, Prdm9 and Runx2 depletion in lA to show their knockdown efficiency. (V) Normalized read counts showing the expression Nfia, Atf3, Stat3, Prdm9 and Runx2 during astroglio- and neurogenesis. (W, X) Immunofluorescence staining of DAPI, GFAP and candidate TFs in ESC-derived astrocytes during different phases of astrogliogenesis, including the progenitor stage (aNPC), D1 (early astrocytes; eA) and D5 (late astrocytes; lA). Scale bar is 100 µm.

Figure S4 (Related to Figure 4):

(A-C) ChIP-seq enrichment of H3K27ac during astrogliogenesis (A) and neurogenesis (B) and H3K4me1 during astrogliogenesis (C) at genomic locations marked by the Nfia motif in eA-specific H3K27ac peaks. (D-F) Same as (A-C) but at genomic locations marked by the Atf3 motif in eA-specific H3K27ac peaks. (G-I) Same as (A-C) but at genomic locations marked by the Lhx2 motif in lN-specific H3K27ac peaks. (J-L) Same as (A-C) but at genomic locations marked by the Runx2 motif in lA-specific H3K27ac peaks. (M-O) Same as (A-C) but at genomic locations marked by the Brn1 motif in lN-specific H3K27ac peaks. (P) Neuronal progenitor cells can either differentiate into astrocytes or neurons. During astroglial differentiation, the Nfia and Atf3 motif sites are specifically enriched among all H3K27ac peaks in eA-during astrogliogenesis and not during neurogenesis. These sites are already primed in aNPC by H3K4me1 mark. Runx2 sites show a gradual gain in H3K27ac enrichment during astrogliogenesis but they do not acquire this mark during neurogenesis. Interestingly, these sites also gain H3K4me1 during astrogliogenesis but its highest levels at Runx2 sites are achieved already at the eA stage. All representations are presented as boxplots with a fold enrichment above the input on the y-axis for H3K27ac during astrogliogenesis (left panels), H3K4me1 during astrogliogenesis (middle panels) and H3K27ac during neurogenesis (right panels) at motif instances of Nfia (eA), Atf3 (eA) and Runx2 (lA).

Figure S5 (Related to Figure 5):

(A) Bright-field images showing the morphology of cells upon depletion of Nfia and Atf3 in eA and Runx2 in lA. Scale bar is 50 µm. (B) Immunofluorescence staining for GFAP and AQP4 upon depletion of Nfia and Atf3 in eA and Runx2 in lA. (C) siRNA ablation of Nfia, Atf3 in eA and Runx2 expression in lA for 1 day and quantification of cell numbers. (D) Rate of apoptosis as determined by Annexin-V staining and flow cytometery in eA transfected with Nfia or Atf3 and lA transfected with Runx2 for 1 day. (E) eA were transfected with Nfia or Atf3 and lA were transfecetd with Runx2 for 1 day and then stained with propidium iodide (PI), and the percentages of cells in G0/G1 and S-G2/M phases of the cell cycle were determined by flow cytometry. (F-H) Boxplot depicting the expression of the downregulated and upregulated genes upon the Nfia and Atf3 KD in eA (F,G) and the Runx2 KD in lA (H) during stages of astroglial and neuronal differentiation. (I-K) Gene set enrichment plot depicting the density of the occurrence of astrocyte genes (genes induced in astrocytes compared to those induced in neurons) and neuronal genes (vice-versa) in Nfia (I), Atf3 (J) and Runx2 (K) deregulated genes.

Figure S6 (Related to Figure 6):

(A) Venn diagram depicting the overlap of genes significantly downregulated by the Nfia and Atf3 KD in eA and their comparison with that of the nearest genes to the H3K27ac peaks during

astrogliogenesis that are enriched with either the Nfia or Atf3 motifs. (B-I) H3K27ac enrichment (B, D-F) and mRNA level (C, G-I) enrichment at the putative Nfia, Atf3 and Runx2 targets sites. (J-M) qPCR following ATAC in aNPC, eA and lA at sites co-bound by Nfia and Atf3 (J) or bound by Nfia (K), Atf3 (L) and Runx2 (M) alone (N-Q). qPCR following ATAC in Nfia- (N, O), Atf3- (N, P) and Runx2-depleted (Q) cells in eA (N-P) and lA (Q) respectively at the putative target regulatory elements.

Figure S7 (Related to Figure 7):

(A) Schematic representation of constructs used for IUE (B) Schematic representation of the IUE experimentation. (C) Immunoblotting after overexpressing the pCIDRE and pCIDRE plasmid encoding Nfia, Atf3 and Runx2 in 293T cells. Flag antibody is used for the immunoblotting. (D) Immunofluorescence of the E18.5 neocortex using the GLAST and TUBB3 antibody. (E) Staining for SATB2 at E18.8, an upper neuronal marker, following overexpression of astroglial TFs by IUE at E15.5. TFs-overexpressing cells are mainly devoid of SATB2 expression.
